# Differential Expression of Extracellular Matrix-Mediated Pathways in Single-Suture Craniosynostosis

**DOI:** 10.1371/journal.pone.0026557

**Published:** 2011-10-19

**Authors:** Brendan D. Stamper, Sarah S. Park, Richard P. Beyer, Theo K. Bammler, Frederico M. Farin, Brig Mecham, Michael L. Cunningham

**Affiliations:** 1 Center for Tissue and Cell Sciences, Seattle Children's Research Institute, Seattle, Washington, United States of America; 2 Department of Environmental and Occupational Health Sciences, University of Washington, Seattle, Washington, United States of America; 3 Sage Bionetworks, Seattle, Washington, United States of America; 4 Division of Craniofacial Medicine and the Department of Pediatrics, University of Washington, Seattle, Washington, United States of America; Naval Research Laboratory, United States of America

## Abstract

Craniosynostosis is a disease defined by premature fusion of one or more cranial sutures. The mechanistic pathology of single-suture craniosynostosis is complex and while a number of genetic biomarkers and environmental predispositions have been identified, in many cases the causes remain controversial and inconclusive. In this study, gene expression data from 199 patients with isolated sagittal (n = 100), unilateral coronal (n = 50), and metopic (n = 49) synostosis are compared against both a control population (n = 50), as well as each other. After controlling for variables contributing to potential bias, *FGF7, SFRP4,* and *VCAM1* emerged as genes associated with single-suture craniosynostosis due to their significantly large changes in gene expression compared to the control population. Pathway analysis implicated focal adhesion and extracellular matrix (ECM)-receptor interaction as differentially regulated gene networks when comparing all cases of single-suture synostosis and controls. Lastly, overall gene expression was found to be highly conserved between coronal and metopic cases, as evidenced by the fact that *WNT2* and *IGFBP2* were the only genes differentially regulated to a significantly large extent in a direct comparison. The identification of genes and gene networks associated with Fgf/Igf/Wnt signaling and ECM-mediated focal adhesion not only support the involvement of biomarkers previously reported to be related to craniosynostosis, but also introduce novel transcripts and pathways that may play critical roles in its pathogenesis.

## Introduction

Craniosynostosis is the pathologic fusion of calvarial bones that is associated with abnormal skull growth and increased intracranial pressure. While the pathogenesis of single-suture craniosynostosis (which occurs in approximately 1/2500 live births) is poorly understood, genetic causes are likely given a 7–10% recurrence rate [1]. However, recurrence rates based on pre-molecular epidemiological data may be upwardly biased because of contamination of nonsyndromic cases with individuals with single gene disorders. The most common form of craniosynostosis involves the fusion of a single suture (85–95%), but cases involving multiple sutures are relatively common (5–15%) [2], [3]. Approximately half of all single-suture craniosynostosis cases involve premature fusion of the sagittal suture, whereas premature fusion of the coronal and metopic sutures occurs in approximately 22% and 15% of cases, respectively. Lambdoid craniosynostosis is very rare, occurring in approximately 2% of all cases [2].

Craniosynostosis can be further categorized into syndromic and non-syndromic forms. Mutations in a number of different genes have been associated with syndromic craniosynostosis such as *FGFR1-3, TWIST1, EFNB1, FBN1, MSX2, RAB23, RECQL4,* and *TGFBR1-2* [4]. In fact, there are over one hundred well-established syndromic forms of craniosynostosis with known modes of inheritance, suggesting that genomic disposition plays an important role in this disease [5]. While multiple reports have identified single gene mutations in nonsyndromic coronal synostosis [6], [7], [8], [9], in general, mutations associated with single-suture synostosis remain elusive and rarely overlap with those causing syndromic forms of the disease [4], [8], [10], [11]. While this evidence suggests a strong genetic component exists for all forms of craniosynostosis, contributions from both genetic and environmental factors likely play a role in premature suture closure for non-syndromic forms of the disease. Results from a number of risk association studies aimed at identifying environmental risk factors related to craniosynostosis have been largely inconclusive [12]; however, evidence for intrauterine head constraint [13], [14], [15], maternal smoking [16], [17], and fertility treatments [18] as predisposing causes does exist.

The fact that a number of environmental and genetic risk factors have been associated with developing craniosynostosis suggests that there is no single gene, factor, or pathway responsible for causing single-suture craniosynostosis. Rather, several independent mechanisms likely lead to the occurrence of several different forms of craniosynostosis, thus complicating the elucidation of these mechanisms [19]. Numerous transcriptomic studies have been performed to gain insight into the pathogenesis of craniosynostosis, however the vast majority analyzed cases of syndromic synostosis [20], [21], [22], [23], or a combination of syndromic and nonsyndromic cases [24], [25]. While these studies have provided great insight into the molecular mechanisms controlling the premature fusion of calvarial sutures in syndromic craniosynostosis, more work is needed to assess gene expression changes in nonsyndromic forms of this disease.

The transcriptomic study presented here is the largest of its kind, and the first to analyze gene expression changes in calvaria osteoblasts as they relate solely to nonsyndromic craniosynostosis. A rich set of transcriptomic data from a panel of well-characterized clinical samples was generated (199 synostosis cases and 50 controls), from which potentially pathogenic changes in gene expression among different forms of single-suture craniosynostosis were identified. In addition, subsequent pathway analysis on the dataset suggested that transcriptomic regulation of genes associated with extracellular matrix (ECM)-mediated focal adhesion play an important role in differentiating patients with craniosynostosis from unaffected individuals.

## Results

### Comparison of suture-based gene expression patterns compared to controls

**Figure 1:**
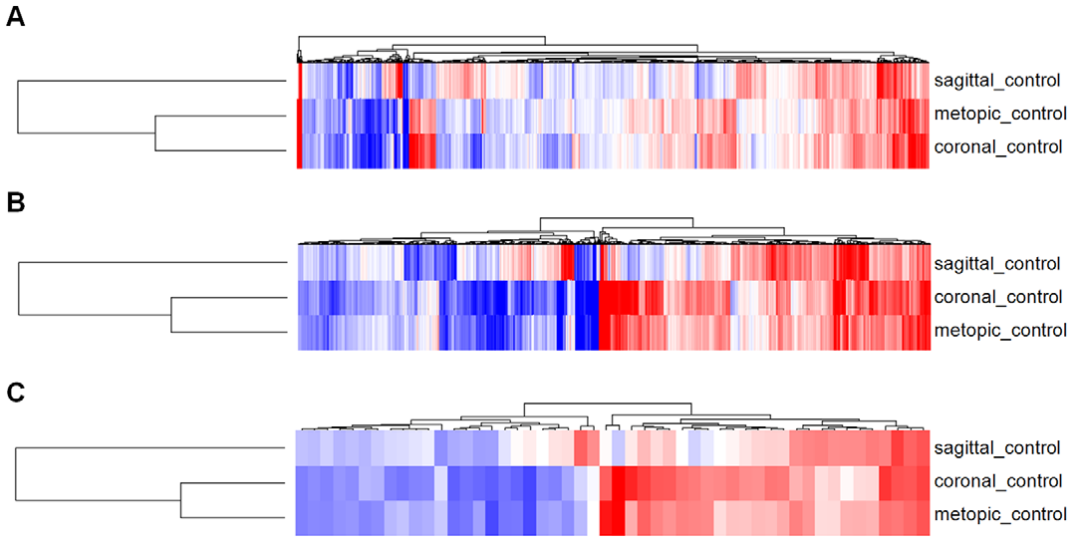
Comparison of Gene Expression patterns between osteoblasts derived from cases of synostosis and control lines. Heatmaps with 2-dimensional hierarchical clustering were generated for the 2000 genes with the highest correlation scores for probe expression (A), and enriched subsets of this gene set where expression levels were considered significant (p<0.05) compared to controls (736 genes) (B), or both significant (p<0.05) and large (|% change| >50) compared to controls (49 genes) (C).

To identify the set of genes that were significantly varying across the sample population, nearly thirty thousand genes were ranked based on their gene information content (GIC) scores, which was defined as the percent variance explained by the first eigengene obtained from a decomposition of the probe-level data for each gene. In other words, high information content genes have consistent probe level expression, meaning that multiple probes within the same gene are changing in a uniform manner. The two thousand genes with the highest GIC scores are listed in Table S1. These genes were then analyzed by 2-dimensional hierarchical clustering, evaluating gene expression patterns among different cases of craniosynostosis compared to controls (Figure 1A). With respect to genes with high GIC scores, the clustering dendrogram is consistent with sagittal cases being distinct from the metopic and coronal cases. Statistical analysis of the gene list revealed that expression levels for 736 of the 2000 (36.8%) were considered significant (p<0.05) when comparing synostosis and control cases (Figure 1B). Again, sagittal cases were distinct from other cases when looking at significant expression changes. The list of significant gene expression changes with high information content was further enriched to include only those changes in gene expression considered to be both significant (p<0.05) and large (|% change| >50) when comparing cases and controls. This comparison identified 49 genes that satisfied these statistical thresholds (Figure 1C). As with previous comparisons (non-significant and significant only), sagittal cases were again distinct from metopic and coronal cases with respect to large and significant changes in gene expression. Interestingly, only the expression of fibroblast growth factor 7 (*FGF7),* vascular cell adhesion molecule 1 *(VCAM1),* and secreted frizzled-related protein 4 *(SFRP4)* were considered to be significant and large in all three cases of single-suture synostosis when compared to controls (Table 1).

**Table 1:** Gene expression consistent in osteoblasts derived from cases of synostosis compared to control lines.

	log2 fold change (% change)			
Gene Symbol	*coronal_control*	*metopic_control*	*sagittal_control*	*all_control*
FGF7	1.01 (101)	0.91 (88)	0.91 (88)	0.89 (85)
VCAM1	0.93 (91)	0.72 (65)	1.04 (106)	0.75 (68)
SFRP4	1.08 (111)	0.76 (69)	0.66 (58)	0.66 (58)

### Comparison of significantly large changes in suture-based gene expression compared to controls

**Figure 2:**
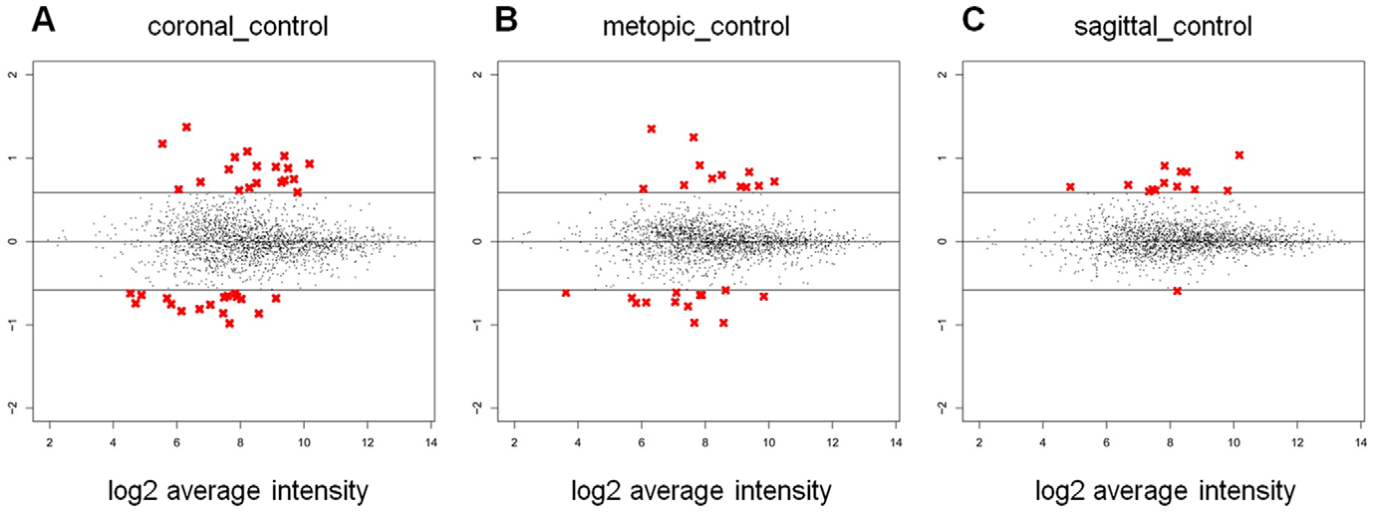
MA-plots highlighting differential gene expression between osteoblasts derived from cases of synostosis and control lines. Genes whose expression was considered to be significant (p<0.05) and large (|% change| >50) are represented by a red “X”, whereas genes whose expression did not meet threshold values are represented by black dots. Comparisons were made between coronal cases and control populations (A), metopic cases and control populations (B), and sagittal cases and control populations (C).

**Figure 3:**
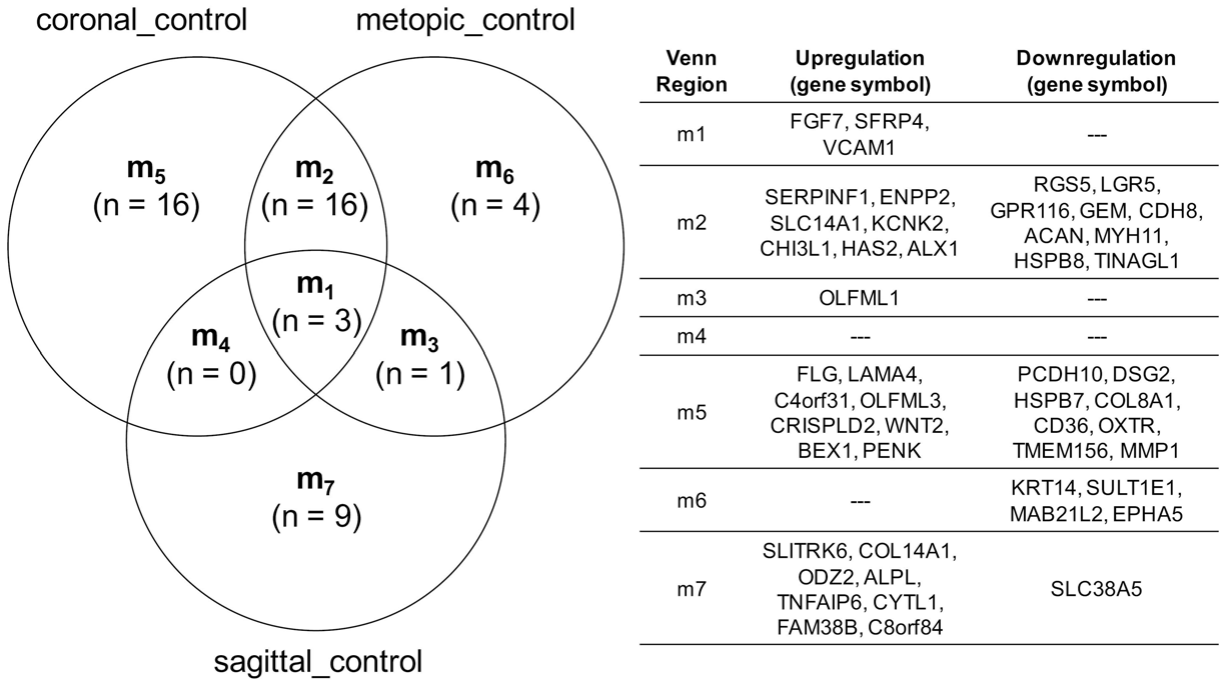
Venn diagram highlighting unique or shared gene sets among different forms of single-suture craniosynostosis. Venn region m1 contains genes shared among all three cases of single-suture synostosis, genes shared between two cases are contained in Venn regions m2, m3, and m4, and genes unique to a specific case are contained in Venn regions m5 (coronal), m6 (metopic), and m7 (sagittal).

Of the 49 gene expression changes considered to be significant and large in at least one or more of the forms of single-suture synostosis (Table S2), 36 were associated with coronal cases, 25 with metopic cases, and 14 with sagittal cases (Figure 2). To fully investigate the relationship between the form of single-suture synostosis and the expression of these genes, Venn diagrams were constructed in order to identify gene sets that were either unique or shared among the cases (Figure 3). Changes in the expression of nineteen of these genes (Venn regions m_1_ and m_2_) were consistent among metopic and coronal cases comprising approximately 79% (for metopic) and 54% (for coronal) of the expression changes considered to be significantly large. Taken together, these results highlight the fact that there are consistent hallmarks of gene expression among osteoblasts derived from cases of synostosis, especially among coronal and metopic cases; however each form of the disease also possesses its own unique expression pattern.

### Direct comparison of gene expression

**Figure 4:**
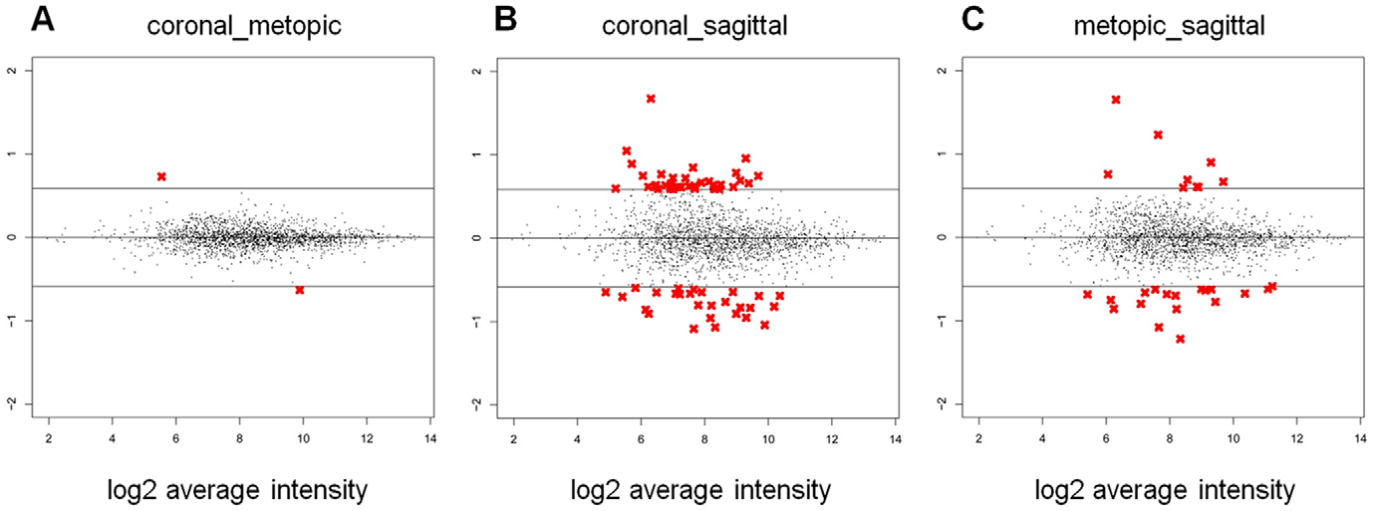
MA plots highlighting differential gene expression by directly comparing osteoblasts derived from cases of synostosis. Genes whose expression was considered to be significant (p<0.05) and large (|% change| >50) are represented by a red “X”, whereas genes whose expression did not meet threshold values are represented by black dots. Comparisons were made between coronal and metopic cases (A), coronal and sagittal cases (B), and metopic and sagittal cases (C).

As gene expression profiles were highly conserved among coronal and metopic cases compared to controls, direct comparisons between osteoblasts derived from these cases of synostosis were investigated (Figure 4). Of the two thousand genes with the highest GIC scores, only two (0.1%) were differentially expressed between coronal and metopic sutures when comparing the cases directly (Figure 4A, Table 2). *WNT2* (wingless-type MMTV integration site family member 2) expression was found to be greater in coronal cases compared to metopic cases; however, *WNT2* expression was significantly higher in both compared to controls (Table 2). In sagittal cases, *WNT2* expression was considered neither large (9% increase) nor significant (p>0.05) compared to controls. Decreased *IGFBP2* (insulin-like growth factor binding protein 2) expression was specific to coronal cases as no significant expression differences were observed between metopic cases and control (Table 2).

**Table 2:** Genes differentially expressed to a significant extent when comparing coronal and metopic cases.

	log2 fold change (% change)	
	*WNT2*	*IGFBP2*
*Coronal_control*	1.17 (125)	−0.56 (−47)
*Metopic_control*	0.44 (36)	0.07 (5)*
*Coronal_metopic*	0.73 (66)	−0.63 (−55)
*Coronal_sagittal*	1.05 (107)	−1.04 (−106)

When directly compared to sagittal cases, both coronal and metopic cases show an increase in the number of genes differentially expressed to a significant and large extent (Figure 4B and 4C). In fact, 22 of these differentially expressed genes were identified in both the coronal versus sagittal and metopic versus sagittal comparisons (Table 3). Furthermore, this subset of genes represents 34% of the total genes in the coronal versus sagittal comparison and 81% of total genes in the metopic versus sagittal comparison. Again, these results highlight highly conserved gene expression patterns in coronal and metopic cases, not only in comparisons to control samples, but also against sagittal craniosynostosis cases directly.

**Table 3:** Differential gene expression consistent among coronal and metopic cases compared to sagittal cases.

	log2 fold change (% change)	
Gene Symbol	*coronal_sagittal*	*metopic_sagittal*
ALX1	1.67 (218)	1.65 (214)
HAS2	0.96 (95)	0.90 (87)
SLC14A1	0.85 (80)	1.23 (135)
CHI3L1	0.75 (68)	0.76 (69)
KCNK2	0.75 (68)	0.67 (59)
CLDN11	0.61 (53)	0.60 (52)
HEY2	−0.65 (−57)	−0.68 (−60)
FAM38B	−0.66 (−58)	−0.62 (−54)
MAB21L2	−0.66 (−58)	−0.80 (−74)
CNTNAP3	−0.67 (−59)	−0.66 (−58)
TGFB2	−0.69 (−61)	−0.67 (−59)
IL26	−0.70 (−62)	−0.68 (−60)
TLR4	−0.81 (−75)	−0.86 (−82)
PCDH10	−0.83 (−78)	−0.64 (−56)
ACTG2	−0.83 (−78)	−0.77 (−71)
LGR5	−0.86 (−82)	−0.75 (−68)
SEMA3D	−0.91 (−88)	−0.86 (−82)
C21orf7	−0.91 (−88)	−0.62 (−54)
PAPPA	−0.95 (−93)	−0.62 (−54)
ASPN	−0.96 (−95)	−0.70 (−62)
C8orf84	−1.07 (−110)	−1.22 (−133)
RGS5	−1.09 (−113)	−1.08 (−111)

### KEGG pathway analysis

**Figure 5:**
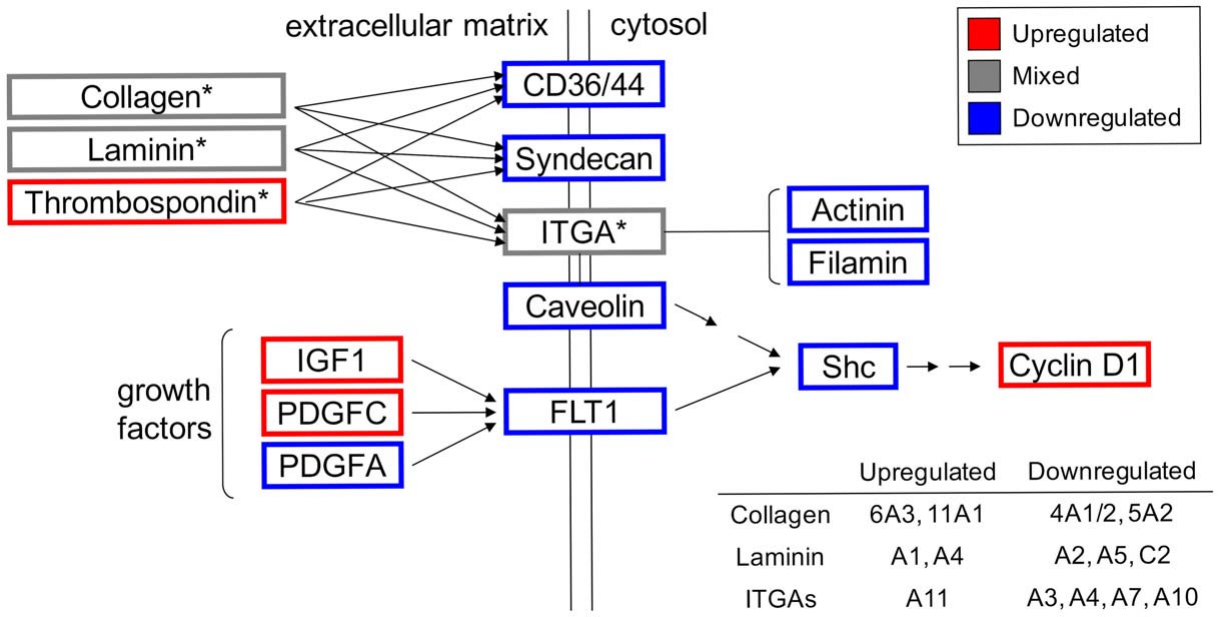
Differential expression of genes related to extracellular matrix-mediated focal adhesion in synostosis cases. Changes in gene expression that were robustly expressed across the population of samples were uploaded into DAVID to identify enriched KEGG pathways potentially affected in craniosynostosis. Genes with significant changes in expression between cases and controls that were related to either focal adhesion or ECM-receptor interactions are mapped in this modified KEGG pathway. Differentially upregulated genes are boxed in red, differentially downregulated genes are boxed in blue, and when up- and downregulated isoforms of the same gene family were observed, mixed expression was assigned (boxed in gray).

Prior analysis of the dataset investigated how similar gene expression patterns were among osteoblasts derived from cases of synostosis, and identified a number of potential gene targets. However, how these changes in expression could affect biological systems was not addressed. To this end, the two thousand genes with the highest GIC scores were uploaded into DAVID in order to identify basic biological pathways associated with genes in our dataset that had consistent changes in expression at the probe level. Using this gene list, focal adhesion and ECM-receptor interaction were the two most significantly implicated pathways (Table S3). In addition, the TGF-beta signaling pathway, regulation of actin cytoskeleton, cell adhesion molecules (CAMs), and gap junction were also identified as significantly enriched pathways (p<0.01). Given that ECM-receptor interactions play a critical role in focal adhesion, genes related to ECM-mediated focal adhesion are of particular interest as potential transcriptomic markers related to craniosynostosis. ECM-mediated focal adhesion is a highly complex interplay between cells and incorporates over fifty known factors [26], therefore only those found to be differentially regulated between synostosis cases and controls are represented in Figure 5. This modified KEGG pathway for ECM-mediated focal adhesion includes the 25 genes associated with focal adhesion, and 19 genes associated with ECM-receptor interactions, that underwent significant changes in expression (p<0.05) when comparing cases and controls. Expression data for these genes can be found in Table S4.

## Discussion

### Transcriptome comparisons among different forms of single-suture craniosynostosis

The existing literature suggests that there is no single pathway that causes craniosynostosis; rather, several independent mechanisms likely lead to craniosynostotic endpoints. While genetic and environmental factors have been implicated in craniosynostosis, the goal of this manuscript was to identify key transcripts associated with single-suture craniosynostosis. While the expression for many genes with high GIC scores changed unilaterally, the clustering dendrograms suggested that sagittal cases were distinct from metopic and coronal cases (Figure 1). The high degree of correlation between coronal and metopic gene expression is clearly visualized by a Venn diagram including the fifty gene expression changes considered significantly large (Figure 3). This diagram highlights the overlap in the expression of nineteen genes shared among coronal and metopic cases (Figure 3, Venn regions m_1_ and m_2_). Perhaps coronal and metopic synostosis share very similar gene expression profiles because these forms of single-suture craniosynostosis are rarer than sagittal synostosis and have fewer root causes. In contrast, sagittal craniosynostosis cases may appear more divergent because there are more root causes, which may or may not be related to its higher incidence in the general population compared to other forms of the disease. It is also possible that differences in the embryonic origin of the calvaria may explain some of the changes in gene expression that were observed, as the frontal and parietal bones are derived from neural crest and paraxial mesoderm, respectively [25], [27].

### FGF7 upregulation in craniosynostosis cases

Even though gene expression in sagittal cases appeared divergent from that of coronal and metopic cases, changes in the expression of three genes were found to be significant and large in all osteoblasts derived from cases of synostosis, *FGF7, VCAM1,* and *SFRP4* (Table 1). Initially, the identification of *FGF7* was most striking, since gain of function mutations in FGF-receptors (FGFRs) cause a number of craniosynostosis syndromes, including Apert, Crouzon, Muenke, and Pfeiffer syndromes [3], [28]. *FGF7* is expressed in loose mesenchyme surrounding the mesenchymal condensation [29] and preferentially activates FGFR2b [30]. However, the S252W and P253R mutations in FGFR2 found in Apert's syndrome allow FGF7-mediated FGFR2c activation [3], [31]. Therefore, upregulation of signaling factors like *FGF7* during mesenchymal condensation may lead to inappropriate ligand-receptor binding, increased mitogenic activity, and thus contribute to skeletal abnormalities related to craniosynostosis.

### *W*NT2/SFRP4 upregulation in craniosynostosis cases

Like *FGF7*, *SFRP4* was identified as a significantly upregulated gene in all osteoblasts derived from cases of synostosis (Table 1). *SFRP4* has been shown to antagonize Wnt activation [32] supporting previous reports that Wnt signaling plays a role in the pathogenesis of craniosynostosis [33], [34], [35], [36]. Furthermore, when a direct comparison between coronal and metopic cases was performed, *WNT2* and *IGFBP2* were the only two genes out of over thirty thousand found to be differentially expressed to a significantly large extent (Figure 4A). The fact that genes associated with Wnt signaling (*WNT2* and *SFRP4*) were identified in these experiments is not surprising due to the fact that Wnt signaling has been implicated not only in genetic disease states related to bone, but also in bone and craniofacial development [37], [38], [39], [40], [41]. In metopic and coronal cases, concurrent *SFRP4* and *WNT2* upregulation may appear counter-intuitive considering *SFRP4* has been shown to antagonize Wnt activation [32], [42], [43]. One possible explanation for this observation is that upregulation of Wnt repressors like *SFRP4,* is a counter-regulatory response to increased *WNT2* expression or vice versa. In fact, simultaneous upregulation in the expression of *WNT2* and *SFRP4* has been previously reported in mouse skin and skeletal muscle [44]. Also, a recent microarray study comparing osteoblast expression from wild-type and Apert syndrome fetuses identified concurrent *WNT2* and *SFRP1* upregulation in the tissues derived from syndromic craniosynostosis cases [22]. Another possible explanation for this scenario is the fact that *WNT2* has been shown to act via noncanonical pathways [45], [46], whereas *SFRP4* has been shown to inhibit canonical Wnt signaling in bone [47]. Based on the complexity of Wnt signaling and potential complications due to tissue-specific functions of specific Wnt isoforms, future studies focusing on the relationship between *WNT2* and *SFRP4* need to be performed in order to elucidate whether concurrent upregulation of these two genes in metopic and coronal cases is related to a compensatory cellular response, canonical/noncanonical Wnt signaling, or crosstalk with unidentified signaling cascades.

### Interplay between Fgf and Wnt signaling

The fact that transcripts associated with Fgf and Wnt signaling were identified as highly differentially regulated in synostosis cases compared to controls, suggests that investigating potential crosstalk mechanisms between these pathways may identify key aspects relating to the pathogenesis of craniosynostosis. Both Fgf and Wnt signaling have been implicated in the determination of mesenchymal cell fate and ossification mechanisms [28], [48], [49], [50]. With respect to Wnt signaling, specificity with respect to canonical and non-canonical pathways is critical, as canonical Wnt signaling appears to repress chondrogenesis, whereas non-canonical Wnt-signaling may promote chondrogenesis via inhibition of canonical pathways [51]. Whether Wnt-mediated chondrogenesis plays a critical role in craniosynostosis is unclear. There is evidence that repression of canonical Wnt signaling prevents premature suture closure [34], [36], however, results from a recent study suggests the interplay between upstream canonical Wnt activity and downstream Fgf signaling is more critical [35]. While signaling mechanisms related to calvarial development and suture maintenance is highly complex, it is evident that crosstalk between Wnt and Fgf signaling pathways plays a key role in mesenchymal cell fate as it relates to premature suture closure.

### The role of ECM-mediated focal adhesion in craniosynostosis

While the identification of individual genes as potential biomarkers for craniosynostosis is useful, it is also important to discover potential network biomarkers for the disease in addition to individual transcripts like *FGF7*, *SFRP4*, and *WNT2*. To this end, pathway analysis was performed to elucidate gene sets in which individual gene expression changes may be smaller in magnitude, however, en masse these genes may heavily implicate specific pathways. When the list consisting of genes with high GIC scores was interrogated using DAVID, two pathways were significantly implicated to a greater degree than all the rest, focal adhesion and ECM-receptor interactions (Table S3). During embryonic development, variations in ECM macromolecule composition influences bone tissue differentiation, so the identification of ECM-mediated focal adhesion as a potential network biomarker for non-syndromic single-suture craniosynostosis is of interest.

Despite some controversy, perturbations in ECM deposition and regulation have been associated with Apert syndrome [21], [52], [53], [54]. In three of these studies, [52], [53], [54] upregulation of ECM components and an increase in matrix mineralization was observed in Apert models, whereas the majority of genes related to cell adhesion and ECM composition was found to be downregulated in the fourth study [21]. In our study, gene expression related to ECM-mediated focal adhesion was mixed, with both up and downregulation of specific ECM components occurring (Figure 5). Although it is difficult to compare single-suture craniosynostosis with syndromic forms of the disease, some of the gene expression changes observed in this study have also been seen in transcriptomic comparisons using tissues from syndromic samples. Most interesting is the fact that one study observed general downregulation of alpha integrin subunits (ITGAs) in syndromic craniosynostosis, except for *ITGA11* [*20*]; exactly what was observed in this study (Figure 5). In another study comparing differential expression during suture fusion from a mix of syndromic and nonsyndromic craniosynostosis cases, *THBS2* and collagen types 2, 3, 4, 6, 8, 10 and 11 were found to be upregulated in unfused sutures [25]. Upregulation *THBS2* and collagen types 6 and 11 were observed in this study as well, alluding to the fact that cartilage-specific gene expression and perturbations to ECM-mediated processes are involved in suture morphogenesis and a common feature in all forms of craniosynostosis.

Finally, identification of ECM-mediated focal adhesion as a candidate network biomarker also substantiates the identification of *VCAM1* and *IGFBP2* as potential individual gene biomarkers for craniosynostosis. Vascular invasion has been characterized as an important step in endochondral ossification [55] and this mechanism of bone formation has been shown to result in premature suture closure [35]. This suggests that perturbations to calvarial vascularization may lead to the disease state. The identification vascular-related transcripts like *VCAM1* (Table 1) and *FLT1* (*VEGFR1*, vascular endothelial growth factor receptor 1) (Figure 5, Table S4) as differentially regulated (p<0.05) between all single-suture synostosis cases and controls, suggests that alterations to vascular components related to ECM-cell interactions may be critical to premature suture closure mechanisms. *FLT1* is a receptor tyrosine kinase (RTK) that plays a key role in focal adhesion-mediated vascular development (Figure 5). Furthermore, mutations in *IGF1R* (insulin-like growth factor 1 receptor), another focal adhesion-related RTK, have been identified as potential causes of single-suture craniosynostosis [56]. Insulin-like growth factor 1 (*IGF1*), a high affinity ligand for IGF1R, was found to be upregulated in all osteoblasts derived from cases of synostosis, albeit only to a significant extent in coronal cases (Figure 5, Table S4). *IGFBP2*, which was found to be downregulated in coronal cases compared to all other treatment conditions (Table 2), is capable of binding to and inhibiting IGF activity. [57], [58], [59]. Therefore, RTK-mediated alterations in focal adhesion, such as IGF signaling (*IGF1, IGFBP2*), vascular invasion (*VCAM1, FLT1*), or other RTK cascades, should be considered potential candidate biomarkers for single-suture craniosynostosis.

### Conclusions

This transcriptomic study has identified a number of potential transcripts and one network biomarker related to craniosynostosis from a rich set of whole genome gene expression data from calvarial osteoblasts derived from a large panel of clinical samples. The results from this study not only identified *FGF7*, *SFRP4*, and *VCAM1 as* novel genetic candidates for the cause of single-suture craniosynostosis like, but also confirmed the involvement of ECM-mediated focal adhesion and Ffg/Wnt/Igf signaling pathways that may contribute its pathogenesis. Furthermore, analysis of transcriptome changes suggest that while the expression of certain genes are consistent among all cases of craniosynostosis, expression patterns for coronal and metopic synostosis are quite similar, whereas gene expression in sagittal cases is more divergent. Future investigations into the regulation of these individual transcripts and gene networks related to the various forms of single-suture craniosynostosis must account for the fact that the mechanistic pathology of this disease is highly complex, likely resulting from a wide array of root causes, both genetic and environmental.

## Materials and Methods

### Ethics statement

Written informed consent was obtained from all participants with single-suture craniosynostosis, whereas a waiver of consent was obtained from the Seattle Children's Hospital institutional review board (IRB) for the anonymous control samples used in this study. This study is HIPAA compliant, and we obtained independent prospective IRB approval from each participating center, including Seattle Children's Hospital, Northwestern University in Chicago, Children's Heath Care of Atlanta, and St. Louis Children's Hospital.

### Participant enrollment

Participants were enrolled as described previously in a prospective, four-center investigation of neurodevelopment among children with single-suture craniosynostosis [60]. Infants were referred to the study at the time of diagnosis by their treating surgeon or pediatrician and were eligible if, at the time of enrollment, they had isolated sagittal, unilateral coronal, metopic, or unilateral lambdoid synostosis confirmed by CT scan. CT scans were performed at each participating center, and de-identified data were sent to Seattle Children's Hospital for diagnosis confirmation. Enrolled cases in the overall study were 84% of those eligible, with distance or time constraints being the major reason for nonparticipation. Lambdoid synostosis cases were excluded from the present study due to insufficient numbers. Exclusion criteria included the presence of major medical or neurological conditions (e.g., cardiac defects, seizure disorders, cerebral palsy, significant health conditions requiring surgical correction, etc.); presence of three or more minor extra-cranial malformations [61]; or presence of other major malformations. Demographic data for the dataset are listed in Table 4.

**Table 4:** Demographic information describing case and control populations.

	n	Average age (mo)	Age range (mo)
**Control**	**50**	**31**	**1–120**
Male	35	24	1**–**96
Female	15	49	1**–**120
**Coronal**	**50**	**11**	**4–24**
Male	18	10	4**–**22
Female	32	11	4**–**24
**Metopic**	**49**	**9**	**3–19**
Male	36	10	4**–**19
Female	13	9	3**–**14
**Sagittal**	**100**	**8**	**2–28**
Male	77	8	3**–**28
Female	23	8	2**–**25

### Osteoblast expansion and culture

Calvaria samples from craniosynostosis cases were obtained from discarded tissues during surgical reconstructive procedures, whereas control calvaria samples were obtained from discarded tissues from anonymous surgical or autopsy specimens. Harvested calvaria samples were then washed with Waymouth's media (Sigma W1625 lot 097K8303) and cleaned of all soft tissue. Calvarial were then sliced into thin 3**–**5mm diameter pieces and placed in 12-well plates (2 pieces per well) containing 2 mL of Waymouth's media supplemented with 2X antibiotic (100X Pen/Strep/Fungizone, Hyclone SV30079.01, lot JUA33955) and 10% FBS (Hyclone SH30070.03, lot ATK33398). Upon reaching confluence, the contents of each 12-well place were trypsinized using 0.05% Trypsin (Hyclone SH30236.02, lot J090511) and passaged into T75 flasks. Again, cells were grown to confluence and passaged into cryogenic vials containing freezing media consisting of 90% fetal bovine serum and 10% DMSO and placed in a liquid nitrogen storage tank. Once ready to use, each osteoblast line was thawed and grown in T25 flasks containing Waymouth's media supplemented with 2X antibiotic (100X Pen/Strep/Fungizone) and 10% FBS. Subsets of the 249 cell lines included 50 controls and 100 sagittal, 50 coronal, and 49 metopic cases with craniosynostosis. Upon reaching 75% confluence, cells were trypsinized using 0.05% Trypsin, counted and passaged at a cell density of 175,000 cells per 25cm^2^. All cells were cultured at 37°C, 5% CO_2_, and 99% humidity. All cell lines were characterized as osteoblasts by alkaline phosphatase staining in 12-well plates. Briefly, one BCIP/NBT tablet (Sigma B5655) was dissolved in 10 mL deionized water, and 500 µL of this solution was added for 30 minutes to each cell line. Representative staining of osteoblasts is shown in Figure S1.

### Cell harvest and RNA isolation

Following the plating of 175,000 cells per 25cm^2^, each osteoblast cell line was once again grown to 75% confluence, photographed for quality control purposes, washed twice with 1X PBS, and trypsinized. An equal volume of media containing FBS was added after trypsin exposure, and cells were centrifuged twice at 200 x g for 10 minutes at 4°C in nuclease free 15ml conical tubes (Corning 430791). Between centrifugation steps, cells were washed once with 1X PBS. Cell pellets were then kept on ice until RNA extraction. For RNA extraction, Roche High Pure miRNA Isolation Kit was used with accordance to the manufacturer's protocol (Roche 050080576001). RNA was stored immediately in −80°C and submitted for microarray processing on dry ice.

### Microarray analysis

RNA integrity was assessed using the Agilent 2100 Bioanalyzer, and only samples passing quality control were analyzed for transcriptomic changes using Affymetrix Human Gene 1.0 ST arrays, on which 28,869 genes are represented. Raw microarray data was processed and analyzed with Bioconductor [62] and normalized with the RMA method as implemented in the Bioconductor affy package [63], [64], [65]. Microarray quality control metrics include the manufacturer's recommended guidelines: (1) visual inspection of probe array images, (2) proper ranking of hybridization and Poly-A controls, and (3) area under the curve values for a receiver operating characteristic plot comparing the positive control and negative control signal values. Other microarray quality control metrics from the Bioconductor affyPLM package [63], [65] were used, including the relative log expression (RLE) values, used to see if expression values are shifted or spread out, and the normalized unscaled standard errors (NUSE), used to see if the variability of genes across arrays is too large. To identify a set of genes whose expression levels vary significantly across the population, singular value decomposition (SVD) of the normalized data for each probe set was performed and the percent variance explained by the 1^st^ singular value was investigated. This value is referred to as the Gene Information Content (GIC). A cutoff for significant GIC scores was defined by permuting the probe-to-probe set map and calculating the percent variance explained for each permuted probe set. This was repeated one thousand times and the cutoff was defined as the 99^th^ percentile of the permuted statistics. Furthermore, any probe set whose observed GIC was less than this value was removed from downstream analyses. All microarray data are MIAME compliant and the raw dataset has been deposited in the MIAME compliant Gene Expression Omnibus (GEO) database under accession number GSE27976 (http://www.ncbi.nlm.nih.gov/geo/).

### Characterization of KGFLP1 expression

Upregulation of keratinocyte growth factor-like protein 1 *(KGFLP1)* was identified as significant and large in all three cases of single-suture synostosis. *KGFLP1* has been characterized as the likely product of a pseudogene with high sequence homology to the C-terminus region of *FGF7* (UniProtKB: Q2TVT4). Because *FGF7* and *KGFLP1* share a high degree of nucleotide sequence identity and several probes that comprise the probe sets corresponding to these transcripts can cross-hybridize, the microarray data was also normalized at the individual probe level and summarized at the exon level using Affymetrix Expression Console software (http://www.affymetrix.com). This approach allowed us to assess the fluorescent signal associated with probes that do not cross-hybridize. For these results it was determined that *FGF7* was in fact cross-hybridizing with the 3′ end probes of *KGFLP1*, and that all probes specific to *KGFLP1* contained in the 5′ end were not differentially expressed.

### DAVID pathway analysis

The initial step in this process was to identify genes that were robustly expressed across the population of samples, which generated a list of two thousand genes ranked by gene information content (GIC) score (Table S1). (GIC) was defined as the percent variance explained by the first eigengene obtained from a decomposition of the probe-level data for each gene. Genes with high GIC scores were uploaded to the online bioinformatics database, DAVID (Database for Annotation, Visualization and Integrated Discovery, http://david.abcc.ncifcrf.gov/) [66], [67]. Using OFFICAL_GENE_SYMBOL as the identifier and *Homo sapiens* as the background, the functional annotation tool was utilized to identify pathways heavily implicated in regards to the enriched dataset.

### Statistical analysis

From the normalized data, genes with significant evidence for differential expression were identified using the limma package [68] in Bioconductor. A mixed effects model was used to investigate the craniosynostosis phenotype while adjusting for age and gender. A blocking variable, microarray processing date, was included as a random effect. P-values were calculated with a modified t-test in conjunction with an empirical Bayes method to moderate the standard errors of the estimated log-fold changes. P-values were adjusted for multiplicity using Bioconductor's implementation of the Benjamini-Hochberg method [69]. The Benjamini-Hochberg method is widely used to calculate false discovery rates for microarray data. Thus, it allows for selecting statistically significant genes while controlling the estimated false discovery rate.

## Supporting Information

Figure S1 **Characterization of primary osteoblast lines.** Representative alkaline phosphatase staining of primary osteoblast lines (10× magnification).
(TIF)

Table S1 **Top 2000 genes with high information content.**
(DOC)

Table S2 **Changes in gene expression considered to be significant and large in at least one form of single-suture craniosynostosis compared to controls.**
(DOC)

Table S3 **Identification of significant KEGG pathways associated with craniosynostosis-related gene expression.**
(DOC)

Table S4 **Genes identified in the dataset related to ECM-mediated focal adhesion with significant changes in expression between cases and controls.**
(DOC)
