# Supplementary figures and images for: Differential Expression of Extracellular Matrix-Mediated Pathways in Single-Suture Craniosynostosis
Source: PLoS One. 2011 Oct 19;6(10):e26557. doi: 10.1371/journal.pone.0026557 (PMC3197523; doi:10.1371/journal.pone.0026557)

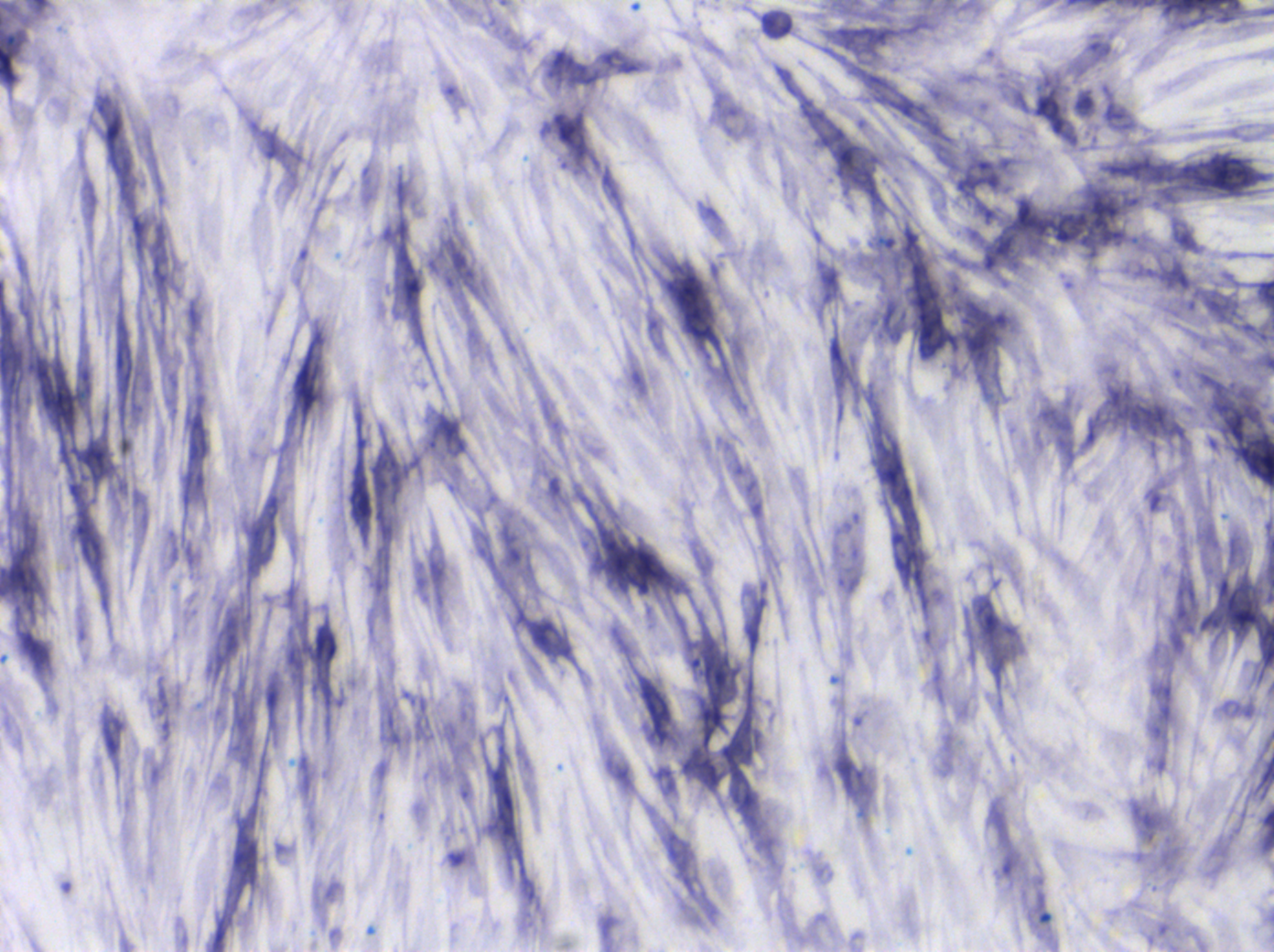

Supplement: Figure S1 — Characterization of primary osteoblast lines. Representative alkaline phosphatase staining of primary osteoblast lines (10× magnification). (TIF) [file pone.0026557.s001.tif]
