# Supplementary material for: Differential Expression of Extracellular Matrix-Mediated Pathways in Single-Suture Craniosynostosis
Source: PLoS One. 2011 Oct 19;6(10):e26557. doi: 10.1371/journal.pone.0026557 (PMC3197523; doi:10.1371/journal.pone.0026557)
Supplement: Table S1 — Top 2000 genes with high information content. (DOC) [file pone.0026557.s002.doc]

**Table S1: Top 2000 genes with high information content**

| **probeID** | **Gene Symbol** | **Information** | **Probes** |
| --- | --- | --- | --- |
| 8176375 | RPS4Y1 | 0.975 | 24 |
| 8176624 | DDX3Y | 0.968 | 42 |
| 8176719 | EIF1AY | 0.966 | 26 |
| 8042788 | ACTG2 | 0.947 | 24 |
| 7920165 | FLG | 0.943 | 21 |
| 7985317 | KIAA1199 | 0.94 | 34 |
| 8133372 | ELN | 0.932 | 48 |
| 8058857 | IGFBP5 | 0.93 | 36 |
| 7944867 | TBRG1 | 0.93 | 4 |
| 8152617 | HAS2 | 0.929 | 28 |
| 8067839 | FGF7P2 | 0.926 | 33 |
| 8097449 | PCDH10 | 0.926 | 43 |
| 8021081 | SLC14A1 | 0.925 | 21 |
| 8107044 | ERAP2 | 0.924 | 28 |
| 7983630 | FGF7 | 0.924 | 24 |
| 7951271 | MMP1 | 0.924 | 31 |
| 8151369 | C8orf84 | 0.923 | 21 |
| 7916493 | PPAP2B | 0.923 | 36 |
| 8152522 | ENPP2 | 0.922 | 30 |
| 7921916 | RGS5 | 0.921 | 35 |
| 8104746 | NPR3 | 0.919 | 36 |
| 8150901 | PENK | 0.919 | 22 |
| 7903358 | VCAM1 | 0.919 | 29 |
| 8150509 | PLAT | 0.917 | 40 |
| 8089145 | ABI3BP | 0.913 | 40 |
| 8146863 | SULF1 | 0.913 | 36 |
| 7972750 | COL4A1 | 0.91 | 61 |
| 7909789 | TGFB2 | 0.909 | 28 |
| 7960744 | C1R | 0.908 | 42 |
| 8174201 | BEX1 | 0.907 | 18 |
| 7938225 | OLFML1 | 0.907 | 32 |
| 8135218 | LRRC17 | 0.906 | 25 |
| 8176578 | USP9Y | 0.905 | 51 |
| 7906919 | RGS4 | 0.904 | 28 |
| 7932254 | ITGA8 | 0.902 | 31 |
| 8177137 | UTY | 0.902 | 47 |
| 8066214 | TGM2 | 0.901 | 40 |
| 8177232 | KDM5D | 0.9 | 38 |
| 8135069 | SERPINE1 | 0.9 | 33 |
| 8066925 | PTGIS | 0.898 | 27 |
| 7951662 | CRYAB | 0.897 | 29 |
| 8128991 | LAMA4 | 0.896 | 53 |
| 8015366 | KRT14 | 0.895 | 16 |
| 8176730 | RPS4Y2 | 0.895 | 28 |
| 8148070 | COL14A1 | 0.893 | 57 |
| 8126798 | GPR116 | 0.893 | 29 |
| 8104663 | CDH6 | 0.892 | 46 |
| 7982377 | GREM1 | 0.891 | 33 |
| 8118890 | SCUBE3 | 0.891 | 27 |
| 8141016 | TFPI2 | 0.89 | 26 |
| 7953603 | C1S | 0.888 | 34 |
| 8083594 | PTX3 | 0.887 | 27 |
| 7899627 | TINAGL1 | 0.886 | 32 |
| 7904158 | HIPK1 | 0.885 | 28 |
| 7985786 | ACAN | 0.884 | 33 |
| 8021635 | SERPINB2 | 0.884 | 36 |
| 8112971 | HAPLN1 | 0.882 | 26 |
| 8097628 | HHIP | 0.881 | 36 |
| 8133876 | CD36 | 0.88 | 43 |
| 8025918 | CNN1 | 0.879 | 26 |
| 7975779 | FOS | 0.879 | 27 |
| 7957452 | ALX1 | 0.878 | 25 |
| 8176698 | CYorf15A | 0.878 | 25 |
| 8064978 | JAG1 | 0.878 | 36 |
| 8129573 | MOXD1 | 0.877 | 25 |
| 8109752 | ODZ2 | 0.877 | 23 |
| 8097773 | MAB21L2 | 0.876 | 22 |
| 8022674 | CDH2 | 0.875 | 43 |
| 8127563 | COL12A1 | 0.875 | 75 |
| 7902074 | LEPR | 0.875 | 48 |
| 7976567 | BDKRB1 | 0.874 | 21 |
| 8145361 | NEFM | 0.874 | 28 |
| 8176655 | NLGN4Y | 0.874 | 18 |
| 7970033 | COL4A2 | 0.873 | 60 |
| 7899480 | SNHG3 | 0.873 | 4 |
| 8003667 | SERPINF1 | 0.872 | 23 |
| 7955613 | KRT7 | 0.871 | 30 |
| 8162394 | ASPN | 0.87 | 33 |
| 7957140 | LGR5 | 0.869 | 42 |
| 8100808 | SULT1E1 | 0.869 | 26 |
| 8015349 | KRT15 | 0.868 | 27 |
| 8105267 | ITGA2 | 0.867 | 33 |
| 7909730 | KCNK2 | 0.865 | 33 |
| 8061136 | PTMAP3 | 0.865 | 4 |
| 8176384 | ZFY | 0.865 | 28 |
| 8106403 | F2RL1 | 0.864 | 27 |
| 7923547 | CHI3L1 | 0.863 | 29 |
| 7980152 | LTBP2 | 0.863 | 45 |
| 8005048 | MYOCD | 0.863 | 37 |
| 7920271 | S100A4 | 0.863 | 15 |
| 7908204 | HMCN1 | 0.862 | 125 |
| 7994237 | LCMT1 | 0.862 | 31 |
| 8013341 | MFAP4 | 0.862 | 24 |
| 8005547 | SNORD3D | 0.861 | 4 |
| 8139087 | SFRP4 | 0.86 | 31 |
| 7965410 | DCN | 0.859 | 36 |
| 8099132 | CYTL1 | 0.858 | 28 |
| 8122222 | PDE7B | 0.858 | 37 |
| 8110932 | SEMA5A | 0.857 | 33 |
| 8152512 | TNFRSF11B | 0.856 | 27 |
| 7997642 | CRISPLD2 | 0.855 | 28 |
| 7940565 | FADS2 | 0.855 | 35 |
| 7915472 | SLC2A1 | 0.855 | 29 |
| 8144786 | SLC7A2 | 0.855 | 32 |
| 8083887 | CLDN11 | 0.854 | 25 |
| 7919815 | CTSK | 0.854 | 27 |
| 8066822 | SULF2 | 0.854 | 30 |
| 8111387 | ADAMTS12 | 0.853 | 32 |
| 8176709 | CYorf15B | 0.853 | 35 |
| 8136200 | CPA4 | 0.852 | 29 |
| 8048205 | IGFBP2 | 0.852 | 28 |
| 7952205 | MCAM | 0.852 | 42 |
| 8131803 | IL6 | 0.851 | 29 |
| 7990345 | SEMA7A | 0.851 | 31 |
| 8151684 | MMP16 | 0.85 | 21 |
| 7965573 | NTN4 | 0.85 | 34 |
| 8104758 | C5orf23 | 0.849 | 24 |
| 8140534 | SEMA3C | 0.849 | 27 |
| 8115490 | ADAM19 | 0.848 | 36 |
| 8097692 | EDNRA | 0.848 | 24 |
| 7934979 | ANKRD1 | 0.847 | 28 |
| 7963880 | ITGA7 | 0.847 | 33 |
| 8123104 | FNDC1 | 0.846 | 46 |
| 8172425 | SLC38A5 | 0.846 | 42 |
| 8006433 | CCL2 | 0.845 | 25 |
| 7952268 | THY1 | 0.845 | 34 |
| 7917875 | F3 | 0.844 | 33 |
| 8040103 | ID2 | 0.844 | 18 |
| 7965873 | IGF1 | 0.844 | 32 |
| 7999674 | MYH11 | 0.844 | 50 |
| 8060940 | C20orf103 | 0.843 | 24 |
| 7966089 | CMKLR1 | 0.843 | 29 |
| 8008885 | MIR21 | 0.843 | 22 |
| 8155460 | CNTNAP3 | 0.841 | 86 |
| 8162373 | OGN | 0.841 | 27 |
| 8168749 | SRPX2 | 0.841 | 25 |
| 8085138 | OXTR | 0.84 | 33 |
| 8088180 | WNT5A | 0.84 | 28 |
| 8112615 | ENC1 | 0.838 | 27 |
| 7971077 | POSTN | 0.837 | 28 |
| 8140668 | SEMA3A | 0.837 | 40 |
| 8149749 | TNFRSF10D | 0.837 | 32 |
| 8113800 | FBN2 | 0.836 | 77 |
| 7953200 | CCND2 | 0.834 | 31 |
| 8138888 | PDE1C | 0.834 | 23 |
| 8139207 | INHBA | 0.833 | 30 |
| 8008237 | ITGA3 | 0.833 | 38 |
| 8120967 | NT5E | 0.833 | 35 |
| 8081810 | GAP43 | 0.832 | 25 |
| 8074980 | GSTT1 | 0.832 | 25 |
| 7908072 | LAMC2 | 0.832 | 33 |
| 8157524 | TLR4 | 0.832 | 36 |
| 8046333 | CYBRD1 | 0.831 | 27 |
| 8089082 | DCBLD2 | 0.831 | 29 |
| 8045835 | GALNT5 | 0.831 | 31 |
| 7970329 | GAS6 | 0.831 | 24 |
| 8121850 | HEY2 | 0.831 | 26 |
| 8015268 | KRT34 | 0.831 | 21 |
| 7965403 | LUM | 0.831 | 26 |
| 8171297 | MID1 | 0.831 | 37 |
| 8112045 | ESM1 | 0.83 | 31 |
| 8122150 | EYA4 | 0.829 | 26 |
| 7903765 | GSTM1 | 0.828 | 21 |
| 8143144 | PTN | 0.828 | 27 |
| 7933772 | ANK3 | 0.827 | 66 |
| 8095728 | EREG | 0.827 | 28 |
| 8154245 | PDCD1LG2 | 0.826 | 27 |
| 8151816 | GEM | 0.824 | 25 |
| 7995681 | MMP2 | 0.824 | 40 |
| 8164580 | PTGES | 0.824 | 22 |
| 7974902 | RHOJ | 0.824 | 32 |
| 8059525 | TM4SF20 | 0.824 | 26 |
| 7918064 | COL11A1 | 0.823 | 74 |
| 8059376 | SERPINE2 | 0.823 | 28 |
| 8102532 | PDE5A | 0.822 | 33 |
| 8129666 | SLC2A12 | 0.822 | 32 |
| 8003298 | SLC7A5P1 | 0.821 | 39 |
| 7966122 | TMEM119 | 0.821 | 29 |
| 7991335 | ANPEP | 0.82 | 27 |
| 8171248 | KAL1 | 0.82 | 36 |
| 8061227 | SLC24A3 | 0.82 | 28 |
| 7942123 | CCND1 | 0.819 | 36 |
| 8148059 | DEPDC6 | 0.819 | 24 |
| 8140579 | CACNA2D1 | 0.818 | 43 |
| 7914342 | FABP3 | 0.818 | 30 |
| 7922976 | PTGS2 | 0.818 | 34 |
| 8163637 | TNC | 0.817 | 38 |
| 8056257 | FAP | 0.816 | 28 |
| 7917503 | GBP3 | 0.816 | 25 |
| 8122099 | ENPP1 | 0.815 | 34 |
| 8106393 | F2R | 0.815 | 36 |
| 8056376 | SCN3A | 0.815 | 38 |
| 8113504 | C5orf13 | 0.814 | 42 |
| 7933194 | CXCL12 | 0.814 | 33 |
| 8114572 | HBEGF | 0.814 | 24 |
| 7898693 | ALPL | 0.813 | 28 |
| 7937335 | IFITM1 | 0.813 | 28 |
| 7963786 | ITGA5 | 0.813 | 31 |
| 8036473 | PPP1R14A | 0.813 | 27 |
| 7954065 | GPRC5A | 0.812 | 24 |
| 8048749 | KCNE4 | 0.812 | 21 |
| 7954090 | EMP1 | 0.811 | 27 |
| 8100578 | EPHA5 | 0.81 | 48 |
| 7934906 | ACTA2 | 0.809 | 30 |
| 8105302 | FST | 0.809 | 25 |
| 8002303 | NQO1 | 0.809 | 30 |
| 8052355 | EFEMP1 | 0.808 | 35 |
| 7923578 | FMOD | 0.808 | 30 |
| 7907222 | PRRX1 | 0.808 | 30 |
| 7924987 | AGT | 0.807 | 28 |
| 7935553 | LOXL4 | 0.807 | 40 |
| 7954293 | PDE3A | 0.806 | 42 |
| 8012475 | MYH10 | 0.805 | 49 |
| 8157487 | PAPPAS | 0.805 | 38 |
| 8149927 | CLU | 0.804 | 45 |
| 8171172 | MXRA5 | 0.804 | 23 |
| 8057677 | SLC40A1 | 0.804 | 34 |
| 8165808 | XG | 0.804 | 22 |
| 7954729 | FGD4 | 0.803 | 26 |
| 7901535 | PODN | 0.803 | 38 |
| 7934997 | PPP1R3C | 0.802 | 27 |
| 7915592 | RNU5D | 0.802 | 24 |
| 8154491 | ADAMTSL1 | 0.801 | 24 |
| 7962579 | AMIGO2 | 0.8 | 35 |
| 8102440 | ARSJ | 0.8 | 34 |
| 8046922 | COL3A1 | 0.8 | 63 |
| 8138824 | SCRN1 | 0.8 | 30 |
| 8102587 | C4orf31 | 0.798 | 27 |
| 8115099 | PDGFRB | 0.798 | 32 |
| 8117120 | ID4 | 0.797 | 22 |
| 7953291 | CD9 | 0.796 | 34 |
| 7981978 | SNORD116-15 | 0.796 | 10 |
| 8150962 | TOX | 0.796 | 31 |
| 8137979 | ACTB | 0.795 | 22 |
| 7900510 | CTPS | 0.795 | 41 |
| 7960947 | A2M | 0.794 | 45 |
| 8131844 | GPNMB | 0.794 | 36 |
| 8139488 | IGFBP3 | 0.794 | 29 |
| 7960919 | MFAP5 | 0.794 | 26 |
| 7986446 | ALDH1A3 | 0.793 | 38 |
| 8073775 | FBLN1 | 0.793 | 30 |
| 8113709 | LOX | 0.793 | 26 |
| 7990054 | UACA | 0.793 | 31 |
| 8170648 | BGN | 0.792 | 32 |
| 8134869 | PCOLCE | 0.792 | 28 |
| 7972239 | SLITRK6 | 0.792 | 36 |
| 8050908 | HADHA | 0.79 | 16 |
| 8115691 | SLIT3 | 0.79 | 47 |
| 7954398 | C12orf39 | 0.789 | 27 |
| 7989501 | CA12 | 0.789 | 34 |
| 7976073 | FLRT2 | 0.789 | 36 |
| 8100298 | OCIAD2 | 0.789 | 25 |
| 7937079 | BNIP3 | 0.788 | 41 |
| 8120932 | PRSS35 | 0.788 | 25 |
| 8152314 | RSPO2 | 0.788 | 35 |
| 8102800 | SLC7A11 | 0.788 | 38 |
| 7977397 | CRIP2 | 0.786 | 28 |
| 7985522 | ADAMTSL3 | 0.785 | 38 |
| 7995783 | MT2A | 0.785 | 12 |
| 8145793 | SNORD13 | 0.785 | 5 |
| 8108370 | EGR1 | 0.784 | 32 |
| 7984813 | ISLR | 0.784 | 25 |
| 7925929 | AKR1C3 | 0.783 | 19 |
| 8049187 | EFHD1 | 0.783 | 27 |
| 7945245 | NTM | 0.783 | 26 |
| 8103399 | PDGFC | 0.782 | 36 |
| 8005171 | TRPV2 | 0.782 | 41 |
| 7964388 | NDUFA4L2 | 0.781 | 28 |
| 8091411 | TM4SF1 | 0.781 | 32 |
| 8115756 | KCNMB1 | 0.78 | 32 |
| 8081431 | ALCAM | 0.779 | 24 |
| 8053231 | LOXL3 | 0.779 | 40 |
| 7961514 | MGP | 0.779 | 29 |
| 8119124 | PI16 | 0.779 | 33 |
| 7912537 | DHRS3 | 0.778 | 36 |
| 8102200 | DKK2 | 0.778 | 31 |
| 7935180 | PDLIM1 | 0.776 | 20 |
| 8111915 | SEPP1 | 0.776 | 25 |
| 7962058 | TMTC1 | 0.776 | 33 |
| 8022283 | FAM38B | 0.775 | 32 |
| 8102831 | C4orf49 | 0.774 | 30 |
| 8081657 | CD200 | 0.774 | 30 |
| 8041781 | EPAS1 | 0.774 | 48 |
| 7931977 | ITIH5 | 0.774 | 30 |
| 8112202 | PLK2 | 0.774 | 38 |
| 8095723 | EPGN | 0.773 | 26 |
| 7953385 | GAPDH | 0.772 | 8 |
| 7969861 | ITGBL1 | 0.772 | 42 |
| 8057744 | STAT1 | 0.772 | 29 |
| 8149774 | LOXL2 | 0.771 | 27 |
| 7903227 | PALMD | 0.771 | 33 |
| 7947512 | PAMR1 | 0.77 | 32 |
| 7908459 | CFH | 0.769 | 26 |
| 8098204 | CPE | 0.769 | 40 |
| 8075310 | LIF | 0.769 | 23 |
| 8163257 | LPAR1 | 0.769 | 26 |
| 7937039 | EBF3 | 0.768 | 40 |
| 8143127 | FAM180A | 0.768 | 26 |
| 8015210 | KRTAP2-3 | 0.768 | 72 |
| 8067985 | NCAM2 | 0.768 | 25 |
| 8041508 | QPCT | 0.768 | 26 |
| 7979824 | ACTN1 | 0.767 | 28 |
| 8156199 | DAPK1 | 0.767 | 30 |
| 7951077 | SESN3 | 0.767 | 26 |
| 8022559 | ANKRD29 | 0.766 | 28 |
| 8007100 | IGFBP4 | 0.766 | 28 |
| 8136983 | OR2A20P | 0.766 | 17 |
| 7928944 | PAPSS2 | 0.766 | 34 |
| 7939383 | PRR5L | 0.766 | 29 |
| 8154135 | SLC1A1 | 0.766 | 29 |
| 7962375 | PRICKLE1 | 0.765 | 23 |
| 8132318 | ANLN | 0.764 | 31 |
| 8154381 | C9orf150 | 0.764 | 33 |
| 8162388 | OMD | 0.764 | 27 |
| 8021301 | RAB27B | 0.764 | 32 |
| 8017885 | ABCA8 | 0.763 | 47 |
| 8045587 | ACVR2A | 0.763 | 35 |
| 8099746 | CCKAR | 0.763 | 23 |
| 8056491 | SCN9A | 0.763 | 30 |
| 8046380 | ITGA6 | 0.762 | 32 |
| 8177277 | RBMY1B | 0.762 | 5 |
| 7932082 | CCDC3 | 0.761 | 35 |
| 8112731 | F2RL2 | 0.761 | 28 |
| 8169240 | PRPS1 | 0.761 | 20 |
| 8044499 | SLC20A1 | 0.761 | 41 |
| 8094778 | UCHL1 | 0.761 | 27 |
| 8069689 | ADAMTS5 | 0.76 | 37 |
| 7969613 | GPC6 | 0.76 | 29 |
| 7921882 | OLFML2B | 0.76 | 42 |
| 8100798 | SULT1B1 | 0.76 | 29 |
| 7946661 | DKK3 | 0.759 | 33 |
| 7980958 | LGMN | 0.759 | 26 |
| 7950555 | LRRC32 | 0.759 | 23 |
| 8157804 | OLFML2A | 0.759 | 38 |
| 8140686 | SEMA3D | 0.759 | 25 |
| 7971590 | CAB39L | 0.758 | 27 |
| 8020903 | GALNT1 | 0.758 | 33 |
| 8076668 | KIAA1644 | 0.758 | 23 |
| 7940028 | SERPING1 | 0.757 | 26 |
| 7936968 | ADAM12 | 0.756 | 32 |
| 8158671 | ASS1 | 0.756 | 24 |
| 7970676 | SHISA2 | 0.756 | 24 |
| 7938390 | ADM | 0.755 | 26 |
| 8140709 | KIAA1324L | 0.755 | 23 |
| 8107100 | RGMB | 0.755 | 30 |
| 8001133 | SHCBP1 | 0.755 | 32 |
| 7920664 | THBS3 | 0.755 | 26 |
| 7923034 | B3GALT2 | 0.754 | 26 |
| 7897801 | RNU5E | 0.754 | 24 |
| 8092707 | LEPREL1 | 0.753 | 26 |
| 7917037 | CRYZ | 0.752 | 30 |
| 8007931 | ITGB3 | 0.752 | 45 |
| 7924071 | KCNH1 | 0.752 | 30 |
| 8147573 | OSR2 | 0.752 | 31 |
| 8008644 | DGKE | 0.751 | 9 |
| 8077970 | FBLN2 | 0.751 | 22 |
| 8018975 | LGALS3BP | 0.751 | 26 |
| 8102792 | PCDH18 | 0.751 | 34 |
| 8045688 | TNFAIP6 | 0.751 | 30 |
| 8106418 | CRHBP | 0.75 | 35 |
| 8111210 | FTHL10 | 0.75 | 13 |
| 8112668 | GCNT4 | 0.75 | 26 |
| 8060897 | PLCB4 | 0.75 | 45 |
| 7971104 | TRPC4 | 0.75 | 27 |
| 8114970 | C5orf46 | 0.749 | 26 |
| 8136248 | MEST | 0.749 | 17 |
| 8104394 | ADCY2 | 0.748 | 35 |
| 8019588 | KRTAP1-5 | 0.748 | 57 |
| 7961142 | OLR1 | 0.748 | 27 |
| 7914467 | SPOCD1 | 0.748 | 29 |
| 8093858 | STK32B | 0.748 | 30 |
| 8014974 | TOP2A | 0.748 | 44 |
| 8069676 | ADAMTS1 | 0.747 | 34 |
| 8115831 | DUSP1 | 0.747 | 32 |
| 8096050 | FGF5 | 0.747 | 20 |
| 8065905 | GDF5 | 0.747 | 28 |
| 8140556 | HGF | 0.747 | 50 |
| 7973067 | PNP | 0.747 | 30 |
| 7962559 | SLC38A4 | 0.747 | 24 |
| 8067029 | KCNG1 | 0.746 | 24 |
| 8034940 | NOTCH3 | 0.746 | 53 |
| 8121225 | GRIK2 | 0.745 | 24 |
| 8132694 | IGFBP1 | 0.745 | 27 |
| 7989647 | KIAA0101 | 0.745 | 27 |
| 8095110 | KIT | 0.745 | 26 |
| 7955589 | NR4A1 | 0.745 | 60 |
| 8103736 | SCRG1 | 0.744 | 26 |
| 7909503 | SERTAD4 | 0.744 | 25 |
| 8083233 | ZIC1 | 0.744 | 30 |
| 8116780 | DSP | 0.743 | 41 |
| 7939052 | FIBIN | 0.743 | 25 |
| 7902541 | IFI44L | 0.743 | 30 |
| 8068083 | C21orf7 | 0.742 | 42 |
| 8021946 | COLEC12 | 0.742 | 28 |
| 7912198 | ENO1 | 0.742 | 17 |
| 8112961 | RPS23 | 0.742 | 26 |
| 8079305 | EXOSC7 | 0.741 | 30 |
| 8124527 | HIST1H1B | 0.741 | 17 |
| 8051583 | CYP1B1 | 0.74 | 36 |
| 7972055 | KCTD12 | 0.74 | 27 |
| 7905329 | MLLT11 | 0.74 | 31 |
| 8033818 | OLFM2 | 0.74 | 26 |
| 8023497 | ATP8B1 | 0.739 | 33 |
| 8081235 | COL8A1 | 0.739 | 35 |
| 8116418 | GFPT2 | 0.739 | 25 |
| 7936494 | GFRA1 | 0.739 | 28 |
| 7925452 | GREM2 | 0.739 | 30 |
| 8174654 | KLHL13 | 0.739 | 28 |
| 8056222 | DPP4 | 0.737 | 30 |
| 8151906 | GDF6 | 0.737 | 29 |
| 8167973 | HEPH | 0.737 | 31 |
| 8163002 | KLF4 | 0.737 | 31 |
| 8134257 | GNG11 | 0.736 | 27 |
| 7945688 | IGF2 | 0.736 | 108 |
| 8057599 | TFPI | 0.736 | 31 |
| 7907160 | ATP1B1 | 0.735 | 26 |
| 7997504 | CDH13 | 0.735 | 31 |
| 8155754 | MAMDC2 | 0.735 | 32 |
| 8046695 | ITGA4 | 0.734 | 38 |
| 8121949 | LAMA2 | 0.734 | 67 |
| 7902023 | RAVER2 | 0.734 | 38 |
| 8030362 | RPL13AP20 | 0.734 | 25 |
| 7999387 | EMP2 | 0.733 | 24 |
| 7976012 | NRXN3 | 0.733 | 49 |
| 8126760 | RCAN2 | 0.733 | 23 |
| 8084891 | FAM43A | 0.732 | 32 |
| 8122634 | SAMD5 | 0.732 | 35 |
| 8142471 | WNT2 | 0.732 | 26 |
| 7981514 | AHNAK2 | 0.731 | 33 |
| 8092726 | CLDN1 | 0.731 | 31 |
| 7947199 | LGR4 | 0.731 | 30 |
| 7954511 | STK38L | 0.731 | 42 |
| 8092849 | ATP13A3 | 0.729 | 40 |
| 8089544 | CCDC80 | 0.729 | 47 |
| 7979133 | NID2 | 0.729 | 31 |
| 8082574 | TRH | 0.729 | 26 |
| 8162179 | GAS1 | 0.728 | 26 |
| 7959102 | HSPB8 | 0.728 | 15 |
| 7908351 | PLA2G4A | 0.728 | 40 |
| 7917850 | ARHGAP29 | 0.727 | 27 |
| 7914127 | IFI6 | 0.727 | 26 |
| 8048541 | DES | 0.726 | 34 |
| 8099524 | LDB2 | 0.726 | 28 |
| 8148435 | WISP1 | 0.726 | 30 |
| 7979307 | DLGAP5 | 0.725 | 23 |
| 7965335 | DUSP6 | 0.724 | 26 |
| 8170119 | FHL1 | 0.724 | 27 |
| 8038890 | HAS1 | 0.724 | 26 |
| 7986757 | NDN | 0.724 | 23 |
| 7927631 | DKK1 | 0.723 | 32 |
| 7965094 | E2F7 | 0.723 | 43 |
| 8121749 | GJA1 | 0.723 | 18 |
| 8001784 | CDH8 | 0.722 | 26 |
| 8119067 | KCTD20 | 0.722 | 33 |
| 7938485 | MICAL2 | 0.722 | 40 |
| 8113491 | STARD4 | 0.721 | 33 |
| 8154692 | TEK | 0.721 | 29 |
| 7954631 | FAR2 | 0.72 | 34 |
| 7964795 | IL26 | 0.72 | 26 |
| 7979505 | SIX1 | 0.72 | 22 |
| 7957260 | GLIPR1 | 0.719 | 30 |
| 8082133 | PDIA5 | 0.719 | 38 |
| 8083569 | TIPARP | 0.719 | 32 |
| 7958253 | C12orf75 | 0.718 | 30 |
| 8035506 | CRLF1 | 0.718 | 29 |
| 8152703 | FBXO32 | 0.718 | 28 |
| 8102468 | PRSS12 | 0.718 | 31 |
| 8141140 | DLX5 | 0.717 | 27 |
| 8124388 | HIST1H3B | 0.717 | 25 |
| 8094476 | TBC1D19 | 0.717 | 24 |
| 8078350 | TGFBR2 | 0.717 | 25 |
| 8065071 | FLRT3 | 0.716 | 32 |
| 8117594 | HIST1H2BM | 0.716 | 17 |
| 8057771 | STAT4 | 0.716 | 30 |
| 7909441 | G0S2 | 0.715 | 25 |
| 7932733 | MKX | 0.715 | 33 |
| 7926545 | PLXDC2 | 0.715 | 39 |
| 7984257 | RNU5A | 0.715 | 4 |
| 7970954 | DCLK1 | 0.714 | 24 |
| 7912692 | HSPB7 | 0.714 | 29 |
| 8138566 | IGF2BP3 | 0.714 | 21 |
| 8105596 | RGS7BP | 0.714 | 24 |
| 8017651 | SMURF2 | 0.714 | 38 |
| 8081298 | GPR128 | 0.713 | 26 |
| 8105229 | ITGA1 | 0.713 | 69 |
| 7951178 | TRPC6 | 0.713 | 33 |
| 7955694 | IGFBP6 | 0.712 | 26 |
| 7925062 | SIPA1L2 | 0.712 | 28 |
| 7965322 | KITLG | 0.711 | 30 |
| 7981346 | RAGE | 0.711 | 29 |
| 7920123 | S100A10 | 0.711 | 28 |
| 8163202 | SVEP1 | 0.711 | 56 |
| 8147837 | ZFPM2 | 0.711 | 25 |
| 8149865 | EBF2 | 0.71 | 24 |
| 8109159 | MIR145 | 0.71 | 25 |
| 8019912 | EMILIN2 | 0.709 | 43 |
| 8091243 | PCOLCE2 | 0.709 | 32 |
| 7987192 | SLC12A6 | 0.709 | 37 |
| 8130867 | THBS2 | 0.709 | 30 |
| 8132250 | BMPER | 0.708 | 48 |
| 7933204 | C10orf10 | 0.708 | 25 |
| 8161892 | GNA14 | 0.708 | 30 |
| 8019578 | KRTAP1-1 | 0.708 | 64 |
| 8137670 | PDGFA | 0.708 | 36 |
| 8080714 | FLNB | 0.707 | 53 |
| 8175539 | LDOC1 | 0.707 | 33 |
| 7939007 | LUZP2 | 0.706 | 30 |
| 7962212 | PKP2 | 0.706 | 36 |
| 7961532 | ARHGDIB | 0.705 | 25 |
| 7917182 | ELTD1 | 0.705 | 40 |
| 7929065 | IFIT1 | 0.705 | 29 |
| 7968015 | TNFRSF19 | 0.705 | 31 |
| 8101828 | TSPAN5 | 0.705 | 24 |
| 8132710 | C7orf69 | 0.704 | 23 |
| 8122637 | SASH1 | 0.703 | 31 |
| 8109490 | SGCD | 0.703 | 37 |
| 8047738 | NRP2 | 0.702 | 38 |
| 8156134 | NTRK2 | 0.702 | 31 |
| 8037374 | PLAUR | 0.701 | 28 |
| 7930980 | PPAPDC1A | 0.701 | 34 |
| 8061564 | ID1 | 0.7 | 32 |
| 7966026 | NUAK1 | 0.7 | 27 |
| 7951807 | CADM1 | 0.699 | 35 |
| 7933509 | ERCC6 | 0.699 | 46 |
| 7909332 | CD55 | 0.698 | 28 |
| 8056890 | CHN1 | 0.698 | 23 |
| 8126946 | RPS17P5 | 0.698 | 20 |
| 7949588 | CD248 | 0.697 | 25 |
| 8098103 | FNIP2 | 0.697 | 25 |
| 8179238 | MICA | 0.697 | 15 |
| 7917912 | DPYD | 0.696 | 30 |
| 8056408 | GALNT3 | 0.696 | 45 |
| 7948332 | LPXN | 0.696 | 26 |
| 8021376 | NEDD4L | 0.696 | 43 |
| 8001197 | NETO2 | 0.696 | 29 |
| 7975390 | SMOC1 | 0.696 | 26 |
| 7956271 | HSD17B6 | 0.695 | 31 |
| 7902553 | IFI44 | 0.695 | 31 |
| 8169233 | KRT18P49 | 0.695 | 4 |
| 8091283 | PLOD2 | 0.695 | 24 |
| 8108217 | TGFBI | 0.695 | 26 |
| 7921909 | C1orf110 | 0.694 | 24 |
| 8015187 | KRTAP1-4 | 0.694 | 10 |
| 7970949 | MAB21L1 | 0.694 | 24 |
| 8114287 | SPOCK1 | 0.693 | 31 |
| 7933469 | ARHGAP22 | 0.692 | 29 |
| 8168622 | KLHL4 | 0.692 | 37 |
| 8094278 | NCAPG | 0.692 | 25 |
| 8149955 | PBK | 0.692 | 30 |
| 8146921 | RDH10 | 0.692 | 29 |
| 7912157 | ERRFI1 | 0.691 | 30 |
| 7928429 | PLAU | 0.691 | 34 |
| 7942941 | CCDC81 | 0.69 | 38 |
| 8124531 | HIST1H3I | 0.69 | 25 |
| 8050548 | LAPTM4A | 0.69 | 29 |
| 7968004 | SGCG | 0.69 | 32 |
| 8035517 | COMP | 0.689 | 24 |
| 8068713 | MX1 | 0.689 | 41 |
| 7980316 | TGFB3 | 0.689 | 26 |
| 8139500 | TNS3 | 0.689 | 43 |
| 7986359 | IGF1R | 0.688 | 30 |
| 7920291 | S100A16 | 0.688 | 26 |
| 8123246 | SLC22A3 | 0.688 | 27 |
| 7968351 | C13orf33 | 0.687 | 25 |
| 8020779 | DSG2 | 0.687 | 36 |
| 8106743 | VCAN | 0.687 | 27 |
| 8097973 | GUCY1B3 | 0.686 | 38 |
| 8172266 | MIR221 | 0.686 | 25 |
| 7946589 | MRVI1 | 0.686 | 27 |
| 8115234 | ANXA6 | 0.685 | 30 |
| 8113073 | ARRDC3 | 0.685 | 29 |
| 8094228 | BST1 | 0.685 | 36 |
| 8019842 | TYMS | 0.685 | 29 |
| 7943711 | C11orf87 | 0.684 | 22 |
| 7974689 | DACT1 | 0.684 | 36 |
| 8131096 | ELFN1 | 0.684 | 26 |
| 8107673 | GRAMD3 | 0.684 | 43 |
| 8080964 | GXYLT2 | 0.684 | 24 |
| 7991406 | PRC1 | 0.684 | 40 |
| 8135576 | TES | 0.684 | 24 |
| 7922889 | IVNS1ABP | 0.683 | 46 |
| 8137010 | CNTNAP2 | 0.682 | 31 |
| 8105908 | OCLN | 0.682 | 33 |
| 7938231 | PPFIBP2 | 0.682 | 34 |
| 7980908 | FBLN5 | 0.681 | 36 |
| 7989985 | ITGA11 | 0.681 | 41 |
| 8048995 | ITM2C | 0.681 | 27 |
| 7979085 | PYGL | 0.681 | 44 |
| 8095585 | SLC4A4 | 0.681 | 33 |
| 8156783 | COL15A1 | 0.68 | 46 |
| 7918825 | CSDE1 | 0.68 | 28 |
| 7922754 | KRT18P28 | 0.68 | 12 |
| 8144802 | PDGFRL | 0.68 | 28 |
| 7999468 | LITAF | 0.679 | 28 |
| 7913655 | ID3 | 0.678 | 29 |
| 7927964 | SRGN | 0.678 | 30 |
| 7963046 | TUBA1B | 0.678 | 8 |
| 7944722 | UBASH3B | 0.678 | 46 |
| 7927681 | BICC1 | 0.677 | 26 |
| 8167560 | GAGE10 | 0.677 | 7 |
| 7936091 | USMG5 | 0.677 | 15 |
| 8023575 | CCBE1 | 0.676 | 33 |
| 8098441 | ODZ3 | 0.676 | 68 |
| 8172280 | SLC9A7 | 0.676 | 37 |
| 8097098 | USP53 | 0.676 | 38 |
| 8103822 | VEGFC | 0.676 | 28 |
| 8088745 | FRMD4B | 0.675 | 27 |
| 7901460 | GPX7 | 0.675 | 28 |
| 7923386 | LMOD1 | 0.675 | 31 |
| 8135488 | LRRN3 | 0.675 | 26 |
| 8088848 | PDZRN3 | 0.675 | 34 |
| 8160452 | CDKN2B | 0.674 | 29 |
| 7916432 | DHCR24 | 0.674 | 26 |
| 8005097 | HS3ST3B1 | 0.674 | 19 |
| 8128565 | POPDC3 | 0.674 | 26 |
| 8089011 | PROS1 | 0.674 | 24 |
| 8144880 | SH2D4A | 0.674 | 39 |
| 8067903 | USP25 | 0.674 | 32 |
| 7904761 | ITGA10 | 0.673 | 42 |
| 8020551 | LAMA3 | 0.673 | 93 |
| 8110084 | MSX2 | 0.673 | 19 |
| 8005839 | TMEM97 | 0.673 | 27 |
| 8104079 | FAT1 | 0.672 | 32 |
| 7899615 | SERINC2 | 0.672 | 35 |
| 8129677 | SGK1 | 0.672 | 54 |
| 8124307 | CMAH | 0.671 | 39 |
| 8114625 | DND1 | 0.671 | 9 |
| 8148049 | NOV | 0.671 | 30 |
| 8104022 | PDLIM3 | 0.671 | 26 |
| 8037283 | PSG4 | 0.671 | 11 |
| 7912887 | MFAP2 | 0.67 | 19 |
| 7913237 | CAMK2N1 | 0.669 | 28 |
| 8069668 | CYYR1 | 0.669 | 30 |
| 8140463 | FGL2 | 0.669 | 23 |
| 8105828 | CCNB1 | 0.668 | 30 |
| 7899187 | DHDDS | 0.668 | 9 |
| 7930894 | GRK5 | 0.668 | 27 |
| 7956018 | RDH5 | 0.668 | 28 |
| 8040223 | RRM2 | 0.668 | 14 |
| 8150537 | SLC20A2 | 0.668 | 39 |
| 8133215 | STAG3L4 | 0.668 | 11 |
| 7918857 | TSPAN2 | 0.668 | 36 |
| 8132557 | AEBP1 | 0.667 | 32 |
| 8007071 | CDC6 | 0.667 | 27 |
| 8095697 | CXCL1 | 0.667 | 23 |
| 8103415 | FAM198B | 0.667 | 34 |
| 8025601 | ICAM1 | 0.667 | 27 |
| 7964602 | LRIG3 | 0.667 | 27 |
| 8090591 | PLXND1 | 0.667 | 62 |
| 8055688 | RND3 | 0.667 | 30 |
| 8067270 | APCDD1L | 0.666 | 34 |
| 8046408 | PDK1 | 0.666 | 45 |
| 8004184 | XAF1 | 0.666 | 36 |
| 8081067 | HTR1F | 0.665 | 25 |
| 7902565 | LPHN2 | 0.665 | 30 |
| 8154100 | VLDLR | 0.665 | 46 |
| 7963567 | KRT8 | 0.664 | 14 |
| 7928308 | DDIT4 | 0.663 | 30 |
| 8000507 | EIF3CL | 0.663 | 54 |
| 8135990 | FLNC | 0.663 | 49 |
| 7983512 | SQRDL | 0.663 | 28 |
| 8130505 | EZR | 0.662 | 26 |
| 8149555 | PSD3 | 0.662 | 25 |
| 7982868 | CHAC1 | 0.661 | 26 |
| 8093191 | DLG1 | 0.661 | 27 |
| 8022118 | EPB41L3 | 0.661 | 39 |
| 7908940 | ATP2B4 | 0.66 | 34 |
| 7965423 | BTG1 | 0.66 | 24 |
| 8053417 | CAPG | 0.66 | 30 |
| 8067969 | CHODL | 0.66 | 33 |
| 8162404 | ECM2 | 0.66 | 46 |
| 8042942 | HK2 | 0.66 | 12 |
| 8151101 | MYBL1 | 0.66 | 38 |
| 8084814 | OSTN | 0.66 | 27 |
| 7929816 | SCD | 0.66 | 14 |
| 8024238 | CIRBP | 0.659 | 33 |
| 8100154 | CORIN | 0.659 | 26 |
| 7954196 | MGST1 | 0.659 | 26 |
| 7950005 | MRGPRF | 0.659 | 25 |
| 7929388 | PLCE1 | 0.659 | 42 |
| 7921099 | CRABP2 | 0.658 | 31 |
| 7948565 | CYBASC3 | 0.658 | 36 |
| 8081386 | NFKBIZ | 0.658 | 44 |
| 8078286 | RARB | 0.658 | 36 |
| 8005200 | SNORD65 | 0.658 | 20 |
| 8140840 | STEAP4 | 0.658 | 27 |
| 8112803 | LHFPL2 | 0.657 | 27 |
| 8094301 | SLIT2 | 0.657 | 41 |
| 7924996 | C1orf198 | 0.656 | 38 |
| 7983969 | CCNB2 | 0.656 | 22 |
| 8026300 | CD97 | 0.656 | 19 |
| 8063187 | EYA2 | 0.656 | 27 |
| 7991234 | MFGE8 | 0.656 | 34 |
| 7909027 | NFASC | 0.656 | 52 |
| 7932985 | NRP1 | 0.656 | 28 |
| 7934278 | P4HA1 | 0.656 | 37 |
| 8113469 | PJA2 | 0.656 | 28 |
| 8029437 | PVR | 0.656 | 32 |
| 8167673 | SNORA11E | 0.656 | 26 |
| 8113433 | EFNA5 | 0.655 | 27 |
| 7995697 | LPCAT2 | 0.655 | 34 |
| 8114767 | PCDH1 | 0.655 | 36 |
| 8004510 | CD68 | 0.654 | 26 |
| 7914880 | COL8A2 | 0.654 | 28 |
| 8116921 | EDN1 | 0.654 | 25 |
| 7922846 | FAM129A | 0.654 | 46 |
| 7915612 | PTCH2 | 0.654 | 22 |
| 8109639 | PTTG1 | 0.654 | 24 |
| 8069178 | ADARB1 | 0.653 | 47 |
| 8005638 | ALDH3A2 | 0.653 | 41 |
| 8131475 | C1GALT1 | 0.653 | 27 |
| 8074991 | GGT5 | 0.653 | 28 |
| 7918558 | KCND3 | 0.653 | 28 |
| 7934570 | KCNMA1 | 0.653 | 54 |
| 7981976 | SNORD116-14 | 0.653 | 15 |
| 7947230 | BDNF | 0.652 | 26 |
| 8055872 | CACNB4 | 0.652 | 35 |
| 8010061 | SPHK1 | 0.652 | 21 |
| 8115851 | STC2 | 0.652 | 36 |
| 8122038 | TMEM200A | 0.652 | 27 |
| 8092177 | NCEH1 | 0.651 | 29 |
| 7902495 | NEXN | 0.651 | 36 |
| 8095080 | PDGFRA | 0.651 | 33 |
| 8091678 | VEPH1 | 0.651 | 24 |
| 7935521 | AVPI1 | 0.65 | 25 |
| 7926875 | BAMBI | 0.65 | 29 |
| 7965090 | CSRP2 | 0.65 | 9 |
| 8100870 | ADAMTS3 | 0.649 | 26 |
| 8111101 | ANKH | 0.649 | 42 |
| 8090098 | MYLK | 0.649 | 72 |
| 8069838 | KRTAP19-5 | 0.648 | 21 |
| 8014115 | MYO1D | 0.648 | 33 |
| 7902127 | SGIP1 | 0.648 | 37 |
| 7977409 | CRIP1 | 0.647 | 54 |
| 7932554 | ARHGAP21 | 0.646 | 54 |
| 7950906 | CTSC | 0.646 | 30 |
| 8026971 | IFI30 | 0.646 | 33 |
| 8138353 | MEOX2 | 0.646 | 32 |
| 8156919 | MURC | 0.646 | 30 |
| 7919028 | TBX15 | 0.646 | 30 |
| 7968883 | C13orf31 | 0.645 | 20 |
| 8059905 | COL6A3 | 0.645 | 55 |
| 8011713 | CXCL16 | 0.645 | 27 |
| 8166469 | SAT1 | 0.645 | 32 |
| 8083090 | ZBTB38 | 0.645 | 25 |
| 8031867 | ZNF135 | 0.645 | 4 |
| 7982358 | ARHGAP11A | 0.644 | 23 |
| 8049471 | CXCR7 | 0.644 | 32 |
| 8090193 | HEG1 | 0.644 | 36 |
| 8035465 | ISYNA1 | 0.644 | 29 |
| 8059413 | DOCK10 | 0.643 | 64 |
| 8010915 | FAM101B | 0.643 | 25 |
| 8022996 | KIAA1632 | 0.643 | 58 |
| 7897620 | PGD | 0.643 | 21 |
| 8010770 | SLC16A3 | 0.643 | 39 |
| 8122724 | ULBP1 | 0.643 | 24 |
| 7902452 | AK5 | 0.642 | 25 |
| 8020955 | MOCOS | 0.642 | 44 |
| 7951351 | PDGFD | 0.642 | 26 |
| 8167287 | PORCN | 0.642 | 32 |
| 7934122 | SAR1A | 0.642 | 25 |
| 8123651 | TUBB2B | 0.642 | 18 |
| 8127201 | COL21A1 | 0.641 | 37 |
| 8147777 | CTHRC1 | 0.641 | 32 |
| 7986329 | NR2F2 | 0.641 | 31 |
| 7946563 | RNF141 | 0.641 | 26 |
| 7953012 | WNT5B | 0.641 | 32 |
| 8022612 | ZNF521 | 0.641 | 30 |
| 7898411 | RNU1-8 | 0.64 | 7 |
| 8098604 | ANKRD37 | 0.639 | 26 |
| 7923086 | ASPM | 0.639 | 46 |
| 8049961 | FBXO25 | 0.639 | 4 |
| 8036151 | HSPB6 | 0.639 | 27 |
| 8015376 | KRT16 | 0.639 | 25 |
| 8109283 | NDST1 | 0.639 | 25 |
| 8056201 | RBMS1 | 0.639 | 19 |
| 8164200 | ANGPTL2 | 0.638 | 32 |
| 8135594 | CAV1 | 0.638 | 20 |
| 8070297 | ERG | 0.638 | 27 |
| 8097086 | MYOZ2 | 0.638 | 32 |
| 8117106 | RNF144B | 0.638 | 38 |
| 8104035 | SORBS2 | 0.638 | 45 |
| 8122807 | AKAP12 | 0.637 | 45 |
| 8085628 | ANKRD28 | 0.637 | 39 |
| 8059854 | ARL4C | 0.637 | 29 |
| 8094101 | GPR78 | 0.637 | 35 |
| 8108301 | KIF20A | 0.637 | 40 |
| 8060997 | SPTLC3 | 0.637 | 47 |
| 8135734 | C7orf58 | 0.636 | 41 |
| 8121685 | DCBLD1 | 0.636 | 42 |
| 7908924 | PRELP | 0.636 | 30 |
| 8068353 | SLC5A3 | 0.636 | 52 |
| 8123920 | ELOVL2 | 0.635 | 32 |
| 8072626 | TIMP3 | 0.635 | 33 |
| 7927710 | CDK1 | 0.634 | 32 |
| 8115543 | EBF1 | 0.634 | 42 |
| 8103722 | HSP90AA6P | 0.633 | 5 |
| 8094789 | LIMCH1 | 0.633 | 50 |
| 8146967 | CRISPLD1 | 0.632 | 29 |
| 7913357 | ECE1 | 0.632 | 46 |
| 8043995 | IL1R1 | 0.632 | 27 |
| 8132860 | EGFR | 0.631 | 39 |
| 7948612 | FADS1 | 0.631 | 36 |
| 8008151 | IGF2BP1 | 0.631 | 46 |
| 8153002 | NDRG1 | 0.631 | 45 |
| 7917649 | TGFBR3 | 0.631 | 24 |
| 8175217 | GPC4 | 0.63 | 30 |
| 8161865 | PRUNE2 | 0.63 | 29 |
| 8106784 | RASA1 | 0.63 | 32 |
| 7957221 | TRHDE | 0.63 | 45 |
| 8123739 | NRN1 | 0.629 | 28 |
| 7906930 | NUF2 | 0.629 | 33 |
| 8138045 | EIF2AK1 | 0.628 | 40 |
| 8147461 | SDC2 | 0.628 | 29 |
| 8168500 | PGK1 | 0.627 | 31 |
| 8060854 | PLCB1 | 0.627 | 41 |
| 8105067 | PTGER4 | 0.627 | 29 |
| 7939839 | PTPRJ | 0.627 | 31 |
| 7928882 | C10orf116 | 0.626 | 29 |
| 8163716 | DBC1 | 0.626 | 32 |
| 8059279 | EPHA4 | 0.626 | 25 |
| 8114805 | FGF1 | 0.626 | 29 |
| 8013465 | KRT16P2 | 0.626 | 32 |
| 8072876 | LGALS1 | 0.626 | 28 |
| 7948794 | LRRN4CL | 0.626 | 25 |
| 8076894 | MLC1 | 0.626 | 26 |
| 7990545 | CSPG4 | 0.625 | 20 |
| 8161520 | PGM5 | 0.625 | 20 |
| 7981996 | SNORD116-24 | 0.625 | 23 |
| 7970763 | FLT1 | 0.624 | 35 |
| 8155327 | ALDH1B1 | 0.623 | 30 |
| 8156290 | CKS2 | 0.622 | 14 |
| 8090433 | MGLL | 0.622 | 40 |
| 8087935 | NT5DC2 | 0.622 | 44 |
| 8006845 | RPL19P12 | 0.622 | 8 |
| 7904726 | TXNIP | 0.622 | 28 |
| 8152606 | SNTB1 | 0.621 | 30 |
| 7957737 | TMPO | 0.621 | 29 |
| 8028524 | ACTN4 | 0.62 | 22 |
| 8146533 | FAM110B | 0.62 | 32 |
| 8016438 | HOXB2 | 0.62 | 31 |
| 8137707 | MIR339 | 0.62 | 22 |
| 8176460 | PRKY | 0.62 | 25 |
| 8008784 | PRR11 | 0.62 | 25 |
| 8098060 | RXFP1 | 0.62 | 26 |
| 8078014 | SLC6A6 | 0.62 | 34 |
| 7918323 | SORT1 | 0.62 | 37 |
| 8164269 | ENG | 0.619 | 44 |
| 8176026 | FLNA | 0.619 | 52 |
| 8024194 | GPX4 | 0.619 | 26 |
| 7925954 | NET1 | 0.619 | 40 |
| 7906954 | PBX1 | 0.619 | 36 |
| 8098581 | SNX25 | 0.619 | 23 |
| 8151032 | GGH | 0.618 | 23 |
| 8178211 | PSMB9 | 0.618 | 31 |
| 7962516 | SLC38A1 | 0.618 | 26 |
| 7964119 | STAT2 | 0.618 | 26 |
| 8030831 | ZNF175 | 0.618 | 25 |
| 8177222 | CD24 | 0.617 | 21 |
| 8114920 | DPYSL3 | 0.617 | 46 |
| 8052733 | PPP3R1 | 0.617 | 4 |
| 8161701 | TMEM2 | 0.617 | 30 |
| 7910111 | EPHX1 | 0.616 | 33 |
| 7943749 | LAYN | 0.616 | 36 |
| 7925320 | NID1 | 0.616 | 26 |
| 8168416 | USMG5P1 | 0.616 | 10 |
| 8056343 | COBLL1 | 0.615 | 31 |
| 7972713 | EFNB2 | 0.615 | 34 |
| 8065576 | FOXS1 | 0.615 | 26 |
| 7942594 | SNORD15B | 0.615 | 25 |
| 8122265 | TNFAIP3 | 0.615 | 38 |
| 8054722 | IL1B | 0.614 | 31 |
| 8155214 | MELK | 0.614 | 45 |
| 7953284 | NTF3 | 0.614 | 26 |
| 8160557 | RPS26P2 | 0.614 | 18 |
| 7984353 | SMAD6 | 0.614 | 37 |
| 8061579 | TPX2 | 0.614 | 28 |
| 8036284 | COX7A1 | 0.613 | 32 |
| 7916898 | DEPDC1 | 0.613 | 18 |
| 7964757 | RBMS1P1 | 0.613 | 11 |
| 7909494 | SYT14 | 0.613 | 29 |
| 7992789 | TNFRSF12A | 0.613 | 28 |
| 7929334 | CEP55 | 0.612 | 30 |
| 7936734 | FGFR2 | 0.612 | 36 |
| 8126839 | TNFRSF21 | 0.612 | 34 |
| 8055624 | ZEB2 | 0.612 | 36 |
| 8116649 | BPHL | 0.611 | 25 |
| 8088866 | CNTN3 | 0.611 | 26 |
| 7937020 | MKI67 | 0.611 | 31 |
| 8047248 | PLCL1 | 0.611 | 29 |
| 8012896 | PMP22 | 0.611 | 27 |
| 7942503 | PPME1 | 0.611 | 33 |
| 8129418 | PTPRK | 0.611 | 45 |
| 7932826 | KIAA1462 | 0.61 | 40 |
| 8105436 | MAP3K1 | 0.61 | 26 |
| 8083779 | SERPINI1 | 0.61 | 32 |
| 8089261 | CBLB | 0.609 | 26 |
| 8102643 | CCNA2 | 0.609 | 33 |
| 8085370 | GSTM5P1 | 0.609 | 8 |
| 7954382 | PYROXD1 | 0.609 | 11 |
| 8058869 | TNS1 | 0.609 | 52 |
| 7909708 | CENPF | 0.608 | 34 |
| 8090214 | SLC12A8 | 0.608 | 44 |
| 8047467 | CDK15 | 0.607 | 36 |
| 8074969 | DDT | 0.607 | 10 |
| 8142345 | DOCK4 | 0.607 | 58 |
| 8091715 | LXN | 0.607 | 32 |
| 7994109 | PLK1 | 0.607 | 24 |
| 7905428 | TUFT1 | 0.607 | 42 |
| 7973850 | AKAP6 | 0.606 | 31 |
| 7954055 | APOLD1 | 0.606 | 25 |
| 8141206 | BAIAP2L1 | 0.606 | 21 |
| 8171921 | DMD | 0.606 | 134 |
| 7912706 | EPHA2 | 0.606 | 40 |
| 8127854 | ME1 | 0.606 | 34 |
| 7965040 | PHLDA1 | 0.606 | 32 |
| 8097080 | SYNPO2 | 0.606 | 20 |
| 8080578 | CACNA2D3 | 0.605 | 43 |
| 7921344 | ELL2 | 0.605 | 9 |
| 7899075 | EXTL1 | 0.605 | 38 |
| 7952325 | HSPA8 | 0.605 | 21 |
| 8068024 | JAM2 | 0.605 | 27 |
| 7946334 | NLRP10 | 0.605 | 23 |
| 8107470 | PTMAP5 | 0.605 | 23 |
| 8091078 | RBP1 | 0.605 | 30 |
| 7977933 | SLC7A8 | 0.605 | 42 |
| 8040514 | UBXN2A | 0.605 | 25 |
| 7931832 | AKR1C2 | 0.604 | 8 |
| 7909568 | DTL | 0.604 | 34 |
| 8042637 | DYSF | 0.604 | 60 |
| 8020183 | IMPA2 | 0.604 | 28 |
| 7926786 | APBB1IP | 0.603 | 46 |
| 7945666 | CTSD | 0.603 | 30 |
| 8165692 | MT-CYB | 0.603 | 25 |
| 8066513 | SDC4 | 0.603 | 27 |
| 7919984 | SELENBP1 | 0.603 | 38 |
| 8104930 | SLC1A3 | 0.603 | 36 |
| 8131600 | TSPAN13 | 0.603 | 36 |
| 8095680 | IL8 | 0.602 | 21 |
| 8123936 | NEDD9 | 0.602 | 37 |
| 8051762 | SLC8A1 | 0.602 | 33 |
| 8172043 | SRPX | 0.602 | 29 |
| 7954653 | TSPAN11 | 0.602 | 24 |
| 8159642 | TUBB2C | 0.602 | 17 |
| 7908543 | NEK7 | 0.601 | 33 |
| 8079966 | SEMA3B | 0.601 | 24 |
| 7988563 | SHC4 | 0.601 | 47 |
| 7993458 | C16orf45 | 0.6 | 26 |
| 7967993 | FGF9 | 0.6 | 28 |
| 8130674 | PDE10A | 0.599 | 29 |
| 7981988 | SNORD116-20 | 0.599 | 15 |
| 8093053 | TFRC | 0.599 | 24 |
| 8167185 | TIMP1 | 0.599 | 27 |
| 7997582 | WFDC1 | 0.599 | 31 |
| 8054254 | AFF3 | 0.598 | 30 |
| 7929052 | IFIT3 | 0.598 | 24 |
| 8067409 | LAMA5 | 0.598 | 98 |
| 8080810 | PTPRG | 0.598 | 45 |
| 8117045 | RBM24 | 0.598 | 26 |
| 8048870 | WDR69 | 0.598 | 34 |
| 8025402 | ANGPTL4 | 0.597 | 31 |
| 8155930 | GCNT1 | 0.597 | 27 |
| 7974835 | PRKCH | 0.597 | 36 |
| 8091698 | SHOX2 | 0.597 | 32 |
| 8111772 | DAB2 | 0.596 | 42 |
| 7980485 | DIO2 | 0.596 | 29 |
| 8116610 | NQO2 | 0.596 | 23 |
| 8059565 | PID1 | 0.596 | 26 |
| 8127932 | TBX18 | 0.596 | 33 |
| 8116272 | ADAMTS2 | 0.595 | 46 |
| 8155898 | PCSK5 | 0.595 | 39 |
| 8070489 | RIPK4 | 0.595 | 32 |
| 7944803 | VWA5A | 0.595 | 28 |
| 8019964 | ARHGAP28 | 0.594 | 31 |
| 7976560 | BDKRB2 | 0.594 | 21 |
| 8120719 | CD109 | 0.594 | 50 |
| 7948630 | FADS3 | 0.594 | 28 |
| 8148317 | MYC | 0.594 | 26 |
| 8158554 | PRRX2 | 0.594 | 30 |
| 8025672 | SLC44A2 | 0.594 | 29 |
| 8047788 | ADAM23 | 0.593 | 29 |
| 8021470 | PMAIP1 | 0.593 | 32 |
| 7939215 | C11orf41 | 0.592 | 25 |
| 7921088 | NES | 0.592 | 27 |
| 8136801 | PRSS2 | 0.592 | 14 |
| 7981084 | SERPINA9 | 0.592 | 34 |
| 8063536 | TFAP2C | 0.592 | 33 |
| 8017210 | AP1S2 | 0.591 | 25 |
| 8072735 | APOL1 | 0.591 | 23 |
| 7917532 | GBP2 | 0.591 | 29 |
| 7929373 | LGI1 | 0.591 | 27 |
| 8099721 | SEL1L3 | 0.591 | 35 |
| 8023727 | DSEL | 0.59 | 28 |
| 8077490 | LMCD1 | 0.59 | 34 |
| 8077366 | LRRN1 | 0.59 | 24 |
| 7984704 | NEO1 | 0.59 | 43 |
| 8060503 | SNORD110 | 0.59 | 25 |
| 7968670 | UFM1 | 0.59 | 19 |
| 7902512 | DNAJB4 | 0.589 | 26 |
| 7903092 | FNBP1L | 0.589 | 30 |
| 8117020 | MYLIP | 0.589 | 30 |
| 8103254 | SFRP2 | 0.589 | 33 |
| 7971866 | DIAPH3 | 0.588 | 36 |
| 8112007 | EMB | 0.588 | 18 |
| 8066393 | JPH2 | 0.588 | 36 |
| 7908694 | NAV1 | 0.588 | 45 |
| 8083968 | NLGN1 | 0.588 | 33 |
| 7897803 | PLOD1 | 0.588 | 46 |
| 7937508 | CD151 | 0.587 | 30 |
| 8160441 | CDKN2A | 0.587 | 20 |
| 8177214 | TTTY14 | 0.587 | 25 |
| 8129458 | ARHGAP18 | 0.586 | 41 |
| 7974404 | CDKN3 | 0.586 | 59 |
| 8018754 | CYGB | 0.586 | 26 |
| 8023995 | FSTL3 | 0.586 | 27 |
| 8049752 | SNED1 | 0.586 | 33 |
| 7999754 | XYLT1 | 0.586 | 29 |
| 8167965 | MSN | 0.585 | 13 |
| 8141829 | POLR2J3 | 0.585 | 44 |
| 8156043 | PSAT1 | 0.585 | 15 |
| 8135601 | MET | 0.584 | 38 |
| 8091306 | PLSCR4 | 0.584 | 31 |
| 7908421 | TROVE2 | 0.584 | 30 |
| 8016094 | GJC1 | 0.583 | 19 |
| 8041383 | LTBP1 | 0.583 | 45 |
| 8147516 | MATN2 | 0.583 | 27 |
| 8051998 | MCFD2 | 0.583 | 23 |
| 8157038 | SLC44A1 | 0.583 | 24 |
| 7984364 | SMAD3 | 0.583 | 37 |
| 7939559 | TSPAN18 | 0.583 | 23 |
| 7999079 | ADCY9 | 0.582 | 34 |
| 8122986 | SNX9 | 0.582 | 26 |
| 7932796 | SVIL | 0.582 | 39 |
| 8018849 | TK1 | 0.582 | 23 |
| 7947681 | ARHGAP1 | 0.581 | 28 |
| 8135587 | CAV2 | 0.581 | 30 |
| 7971150 | LHFP | 0.581 | 29 |
| 8091648 | SSR3 | 0.581 | 22 |
| 8047300 | AOX1 | 0.58 | 39 |
| 7965941 | GLT8D2 | 0.58 | 26 |
| 8102950 | INPP4B | 0.58 | 30 |
| 7938777 | LDHA | 0.58 | 22 |
| 7899029 | MAN1C1 | 0.58 | 42 |
| 8103834 | AGA | 0.579 | 26 |
| 7982757 | CASC5 | 0.579 | 49 |
| 7905220 | ECM1 | 0.579 | 27 |
| 8139212 | GLI3 | 0.578 | 44 |
| 8076455 | RRP7B | 0.578 | 14 |
| 8140967 | SAMD9 | 0.578 | 30 |
| 8072710 | APOL6 | 0.577 | 34 |
| 8037537 | ERCC2 | 0.577 | 25 |
| 8071809 | GSTT2B | 0.577 | 32 |
| 8085867 | NEK10 | 0.577 | 48 |
| 8038407 | RRAS | 0.577 | 25 |
| 8133106 | SNORA22 | 0.577 | 8 |
| 8106170 | TMEM171 | 0.577 | 25 |
| 7928189 | UNC5B | 0.577 | 40 |
| 7952309 | BLID | 0.576 | 25 |
| 8156228 | CTSL1 | 0.576 | 24 |
| 7916112 | RAB3B | 0.576 | 28 |
| 8163896 | STOM | 0.576 | 26 |
| 8157216 | UGCG | 0.576 | 35 |
| 8110618 | ARPP19 | 0.575 | 4 |
| 7965359 | ATP2B1 | 0.575 | 29 |
| 8145365 | DOCK5 | 0.575 | 70 |
| 7980680 | FOXN3 | 0.574 | 16 |
| 7991224 | HAPLN3 | 0.574 | 32 |
| 8179704 | IER3 | 0.574 | 28 |
| 8056151 | PLA2R1 | 0.574 | 43 |
| 8058390 | RAPH1 | 0.574 | 31 |
| 7916862 | WLS | 0.574 | 42 |
| 7993478 | ABCC1 | 0.573 | 34 |
| 8008609 | ANKFN1 | 0.573 | 38 |
| 8151993 | COX6C | 0.573 | 18 |
| 7958019 | DRAM1 | 0.573 | 29 |
| 8144436 | FAM90A15 | 0.573 | 41 |
| 8089112 | FILIP1L | 0.573 | 46 |
| 8057517 | NCKAP1 | 0.573 | 37 |
| 8092409 | PARL | 0.573 | 20 |
| 7974471 | UBE2L7P | 0.573 | 12 |
| 8169009 | BEX4 | 0.572 | 26 |
| 8004497 | EIF4A1P4 | 0.572 | 16 |
| 8124492 | HIST1H2BK | 0.572 | 9 |
| 8124437 | HIST1H3F | 0.572 | 21 |
| 8132503 | STK17A | 0.572 | 34 |
| 8118734 | ITPR3 | 0.571 | 62 |
| 8142997 | PLXNA4 | 0.571 | 51 |
| 7929593 | RPL13AP5 | 0.571 | 10 |
| 8131614 | AHR | 0.57 | 40 |
| 7937330 | IFITM2 | 0.57 | 15 |
| 8161648 | KLF9 | 0.57 | 32 |
| 8006820 | LASP1 | 0.57 | 26 |
| 7958884 | OAS1 | 0.57 | 27 |
| 8107270 | TSLP | 0.57 | 30 |
| 8083494 | MME | 0.569 | 31 |
| 8090018 | PARP9 | 0.569 | 32 |
| 8139264 | POLR2J | 0.569 | 22 |
| 8035829 | RPL34 | 0.569 | 12 |
| 8067167 | AURKA | 0.568 | 21 |
| 8124534 | HIST1H4L | 0.568 | 26 |
| 8055314 | LYPD1 | 0.568 | 26 |
| 8042356 | MEIS1 | 0.568 | 40 |
| 7927732 | ARID5B | 0.567 | 37 |
| 8168873 | ARMCX6 | 0.567 | 4 |
| 8009353 | PITPNC1 | 0.567 | 33 |
| 8042439 | ANTXR1 | 0.566 | 31 |
| 8005679 | CCDC144A | 0.566 | 20 |
| 7925525 | CEP170 | 0.566 | 8 |
| 8041206 | LBH | 0.566 | 30 |
| 7948987 | PLA2G16 | 0.566 | 25 |
| 7978407 | PRKD1 | 0.566 | 29 |
| 8030980 | ZNF845 | 0.566 | 5 |
| 8109093 | ABLIM3 | 0.565 | 30 |
| 8148448 | KHDRBS3 | 0.565 | 33 |
| 7995813 | MT1DP | 0.565 | 26 |
| 8157650 | PTGS1 | 0.565 | 34 |
| 8023598 | RNF152 | 0.565 | 25 |
| 7976812 | SNORD113-4 | 0.565 | 22 |
| 8027002 | GDF15 | 0.564 | 24 |
| 7951535 | KDELC2 | 0.564 | 31 |
| 8091972 | MECOM | 0.564 | 44 |
| 7904695 | SEC22B | 0.564 | 26 |
| 8156826 | TGFBR1 | 0.564 | 33 |
| 8031825 | ZNF552 | 0.564 | 4 |
| 8147756 | BAALC | 0.563 | 27 |
| 7943413 | BIRC3 | 0.563 | 34 |
| 8020455 | GATA6 | 0.563 | 34 |
| 8117377 | HIST1H1E | 0.563 | 19 |
| 7953274 | KCNA1 | 0.563 | 27 |
| 8032909 | PLIN3 | 0.563 | 28 |
| 8054580 | BUB1 | 0.562 | 30 |
| 8117368 | HIST1H4C | 0.562 | 21 |
| 7956867 | HMGA2 | 0.562 | 31 |
| 8108905 | KCTD16 | 0.562 | 28 |
| 8160138 | NFIB | 0.562 | 36 |
| 7927606 | PRKG1 | 0.562 | 25 |
| 7922416 | SNORD76 | 0.562 | 25 |
| 8023528 | ALPK2 | 0.561 | 28 |
| 7981427 | CKB | 0.561 | 32 |
| 8127778 | FAM46A | 0.561 | 30 |
| 8156770 | GALNT12 | 0.561 | 30 |
| 7916986 | NEGR1 | 0.561 | 31 |
| 8120043 | RUNX2 | 0.561 | 27 |
| 8071737 | SLC2A11 | 0.561 | 29 |
| 7902435 | TPI1P1 | 0.561 | 11 |
| 8129254 | MAN1A1 | 0.56 | 42 |
| 8068697 | MX2 | 0.56 | 32 |
| 7950838 | PICALM | 0.56 | 25 |
| 7933872 | EGR2 | 0.559 | 32 |
| 8097288 | FAT4 | 0.559 | 70 |
| 7919642 | HIST2H2AB | 0.559 | 24 |
| 7914525 | MARCKSL1 | 0.559 | 26 |
| 7937148 | MIR202 | 0.559 | 25 |
| 8058273 | MPP4 | 0.559 | 25 |
| 8129880 | PERP | 0.559 | 22 |
| 7902400 | RABGGTB | 0.559 | 17 |
| 8072413 | SMTN | 0.559 | 28 |
| 8117165 | SOX4 | 0.559 | 37 |
| 7953532 | ENO2 | 0.558 | 32 |
| 8175666 | GABRE | 0.558 | 46 |
| 8020164 | GNAL | 0.558 | 22 |
| 8037005 | TGFB1 | 0.558 | 33 |
| 8116818 | BMP6 | 0.557 | 30 |
| 8084880 | HES1 | 0.557 | 27 |
| 8102560 | MAD2L1 | 0.557 | 33 |
| 8142981 | PODXL | 0.557 | 30 |
| 7982187 | APBA2 | 0.556 | 38 |
| 8112376 | CENPK | 0.556 | 26 |
| 8073015 | KDELR3 | 0.556 | 26 |
| 8075728 | MYH9 | 0.556 | 58 |
| 7978123 | PSME2 | 0.556 | 11 |
| 7974366 | PTGER2 | 0.556 | 30 |
| 8014233 | SLFN11 | 0.556 | 25 |
| 7984475 | CORO2B | 0.555 | 24 |
| 8086615 | LRRC2 | 0.555 | 34 |
| 7943892 | NCAM1 | 0.555 | 25 |
| 8085914 | SLC4A7 | 0.555 | 39 |
| 7982086 | SNORD115-40 | 0.555 | 19 |
| 8047272 | SPATS2L | 0.555 | 34 |
| 8113773 | ALDH7A1 | 0.554 | 9 |
| 8063458 | DOK5 | 0.554 | 30 |
| 7930208 | INA | 0.554 | 23 |
| 7995797 | MT1L | 0.554 | 18 |
| 8045247 | PLEKHB2 | 0.554 | 20 |
| 8098414 | SPCS3 | 0.554 | 22 |
| 8138487 | ASS1P11 | 0.553 | 15 |
| 7948420 | FABP5 | 0.553 | 65 |
| 7949412 | LTBP3 | 0.553 | 29 |
| 7898057 | PDPN | 0.553 | 30 |
| 7904293 | PTGFRN | 0.553 | 35 |
| 8116952 | RNU1-11P | 0.553 | 4 |
| 8127987 | SNHG5 | 0.553 | 25 |
| 7979241 | BMP4 | 0.552 | 31 |
| 8138489 | CDCA7L | 0.552 | 31 |
| 7918379 | GSTM3 | 0.552 | 33 |
| 7974851 | HIF1A | 0.552 | 40 |
| 7906264 | PEAR1 | 0.552 | 27 |
| 8123989 | RANBP9 | 0.552 | 37 |
| 8094460 | RBPJ | 0.552 | 23 |
| 8034512 | SNORD41 | 0.552 | 25 |
| 8152355 | SYBU | 0.552 | 28 |
| 8112342 | ADAMTS6 | 0.551 | 48 |
| 7925939 | AKR1C4 | 0.551 | 22 |
| 8113214 | GLRX | 0.551 | 17 |
| 7929258 | KIF11 | 0.551 | 26 |
| 8168146 | KIF4A | 0.551 | 15 |
| 8135568 | MDFIC | 0.551 | 36 |
| 7950933 | NOX4 | 0.551 | 19 |
| 7982889 | NUSAP1 | 0.55 | 33 |
| 8098576 | SLC25A4 | 0.55 | 25 |
| 8109830 | CCDC99 | 0.549 | 36 |
| 7898263 | FBLIM1 | 0.549 | 25 |
| 7974316 | FRMD6 | 0.549 | 46 |
| 7959893 | GPR133 | 0.549 | 35 |
| 8132092 | INMT | 0.549 | 28 |
| 7945262 | JAM3 | 0.549 | 28 |
| 8087337 | LAMB2 | 0.549 | 43 |
| 7899005 | TMEM50A | 0.549 | 15 |
| 7930870 | TOMM22 | 0.549 | 4 |
| 8088680 | C3orf64 | 0.548 | 26 |
| 7908488 | CFHR1 | 0.548 | 10 |
| 8046895 | FAM171B | 0.548 | 28 |
| 7941936 | GSTP1 | 0.548 | 28 |
| 8077499 | LOH3CR2A | 0.548 | 24 |
| 7950374 | P4HA3 | 0.548 | 36 |
| 7979033 | SAV1 | 0.548 | 23 |
| 8098195 | SC4MOL | 0.548 | 25 |
| 8085556 | SH3BP5 | 0.548 | 34 |
| 8178090 | SNORD52 | 0.548 | 43 |
| 7966829 | WSB2 | 0.548 | 26 |
| 8138067 | CYTH3 | 0.547 | 36 |
| 8124430 | HIST1H1D | 0.547 | 20 |
| 7952145 | HYOU1 | 0.547 | 38 |
| 8094870 | SHISA3 | 0.547 | 20 |
| 7930631 | TDRD1 | 0.547 | 42 |
| 8160637 | B4GALT1 | 0.546 | 30 |
| 8032371 | FAM108A4 | 0.546 | 9 |
| 7984540 | KIF23 | 0.546 | 25 |
| 8095303 | LPHN3 | 0.546 | 32 |
| 8081548 | PVRL3 | 0.546 | 40 |
| 8078529 | STAC | 0.546 | 33 |
| 8065353 | THBD | 0.546 | 31 |
| 8057578 | CALCRL | 0.545 | 39 |
| 8149574 | CSGALNACT1 | 0.545 | 34 |
| 8089299 | CD47 | 0.544 | 33 |
| 8093494 | CRIPAK | 0.544 | 17 |
| 7958262 | TCP11L2 | 0.544 | 32 |
| 7930194 | CNNM2 | 0.543 | 40 |
| 7964460 | DDIT3 | 0.543 | 25 |
| 8143154 | DGKI | 0.543 | 39 |
| 8121601 | FAM26E | 0.543 | 30 |
| 8050302 | ROCK2 | 0.543 | 36 |
| 8172154 | RPS2 | 0.543 | 13 |
| 7979455 | RTN1 | 0.543 | 36 |
| 8019486 | SECTM1 | 0.543 | 29 |
| 8021047 | SETBP1 | 0.543 | 24 |
| 8017704 | AMZ2P1 | 0.542 | 12 |
| 7975324 | GALNTL1 | 0.542 | 22 |
| 7901601 | MRPL37 | 0.542 | 32 |
| 8120249 | RN7SK | 0.542 | 34 |
| 8045889 | TANC1 | 0.542 | 40 |
| 8029693 | FOSB | 0.541 | 29 |
| 8111670 | GDNF | 0.541 | 25 |
| 8127087 | GSTA3 | 0.541 | 18 |
| 8075126 | MN1 | 0.541 | 40 |
| 8004506 | SNORA48 | 0.541 | 25 |
| 7982094 | SNORD115-44 | 0.541 | 12 |
| 8170635 | ZNF275 | 0.541 | 52 |
| 7957386 | ACSS3 | 0.54 | 39 |
| 7954021 | CREBL2 | 0.54 | 27 |
| 8111941 | HMGCS1 | 0.54 | 34 |
| 7995829 | MT1H | 0.54 | 16 |
| 8173551 | PHKA1 | 0.54 | 29 |
| 8148315 | POU5F1B | 0.54 | 13 |
| 8139879 | SKP1P1 | 0.54 | 6 |
| 8056572 | SPC25 | 0.54 | 30 |
| 8096959 | ANK2 | 0.539 | 73 |
| 8088642 | LRIG1 | 0.539 | 27 |
| 8092185 | RNU4-4P | 0.539 | 15 |
| 7970569 | SACS | 0.539 | 33 |
| 7948904 | SNORD26 | 0.539 | 25 |
| 8069269 | COL6A1 | 0.538 | 35 |
| 7949971 | CPT1A | 0.538 | 47 |
| 7902290 | CTH | 0.538 | 30 |
| 8133721 | HSPB1 | 0.538 | 18 |
| 8037236 | PSG8 | 0.538 | 9 |
| 8020110 | RAB31 | 0.538 | 27 |
| 8166402 | SMS | 0.538 | 9 |
| 8078600 | TCEA1 | 0.538 | 30 |
| 8145470 | DPYSL2 | 0.537 | 39 |
| 7969438 | LMO7 | 0.537 | 45 |
| 8000636 | NPIPL3 | 0.537 | 10 |
| 8099850 | TMEM156 | 0.537 | 36 |
| 8040365 | TRIB2 | 0.537 | 26 |
| 8140955 | CDK6 | 0.536 | 40 |
| 8107850 | CHSY3 | 0.536 | 20 |
| 8050160 | MBOAT2 | 0.536 | 35 |
| 8019857 | NDC80 | 0.536 | 35 |
| 7918869 | NGF | 0.536 | 27 |
| 7902038 | AK3L2 | 0.535 | 8 |
| 7943162 | C11orf54 | 0.535 | 25 |
| 7950067 | DHCR7 | 0.535 | 31 |
| 8007141 | EIF1 | 0.535 | 18 |
| 8069770 | GRIK1 | 0.535 | 44 |
| 7938880 | HTATIP2 | 0.535 | 26 |
| 8016708 | LRRC59 | 0.535 | 22 |
| 8090044 | SEMA5B | 0.535 | 37 |
| 8112865 | SERINC5 | 0.535 | 29 |
| 8080685 | SLMAP | 0.535 | 32 |
| 8043100 | TMSB10 | 0.535 | 18 |
| 8001457 | CES1 | 0.534 | 9 |
| 7910923 | FMN2 | 0.534 | 39 |
| 8054479 | MALL | 0.534 | 27 |
| 8136662 | MGAM | 0.534 | 47 |
| 8024003 | PALM | 0.534 | 32 |
| 8163328 | PTGR1 | 0.534 | 24 |
| 8070182 | RCAN1 | 0.534 | 26 |
| 7948894 | RNU2-2 | 0.534 | 12 |
| 8098246 | ANXA10 | 0.533 | 31 |
| 8097513 | MGST2 | 0.533 | 26 |
| 7932765 | MPP7 | 0.533 | 42 |
| 8164215 | RPL12 | 0.533 | 23 |
| 7938370 | SWAP70 | 0.533 | 34 |
| 7958174 | TXNRD1 | 0.533 | 27 |
| 8074606 | USP18 | 0.533 | 29 |
| 8054702 | CKAP2L | 0.532 | 26 |
| 7930413 | DUSP5 | 0.532 | 31 |
| 7912520 | NPPB | 0.532 | 28 |
| 7963157 | RACGAP1P | 0.532 | 34 |
| 7997942 | CPNE7 | 0.531 | 44 |
| 7999553 | CPPED1 | 0.531 | 29 |
| 8116520 | GNB2L1 | 0.531 | 23 |
| 8094625 | KLHL5 | 0.531 | 29 |
| 8148553 | LY6K | 0.531 | 29 |
| 8059783 | NGEF | 0.531 | 40 |
| 8081590 | PHLDB2 | 0.531 | 29 |
| 7923991 | PLXNA2 | 0.531 | 64 |
| 8106986 | RHOBTB3 | 0.531 | 32 |
| 7915590 | RNU5F | 0.531 | 25 |
| 7974816 | SLC38A6 | 0.531 | 37 |
| 8161044 | TPM2 | 0.531 | 30 |
| 8030007 | EMP3 | 0.53 | 32 |
| 7908650 | IGFN1 | 0.53 | 26 |
| 8107234 | MAN2A1 | 0.53 | 33 |
| 8002403 | MTSS1L | 0.53 | 39 |
| 7898939 | NIPAL3 | 0.53 | 23 |
| 8122426 | PHACTR2 | 0.53 | 36 |
| 8143772 | RARRES2 | 0.53 | 27 |
| 8042310 | SLC1A4 | 0.53 | 33 |
| 8162759 | TBC1D2 | 0.53 | 24 |
| 7930380 | ADD3 | 0.529 | 39 |
| 7989037 | CCPG1 | 0.529 | 30 |
| 8048026 | CPS1 | 0.529 | 46 |
| 8037408 | KCNN4 | 0.529 | 42 |
| 8092541 | LIPH | 0.529 | 30 |
| 8147221 | OSGIN2 | 0.529 | 32 |
| 8041644 | PLEKHH2 | 0.529 | 41 |
| 8015460 | ACLY | 0.528 | 30 |
| 7945182 | APLP2 | 0.528 | 29 |
| 7917707 | EVI5 | 0.528 | 26 |
| 7997740 | MAP1LC3B | 0.528 | 13 |
| 7928679 | PLAC9 | 0.528 | 18 |
| 7964872 | PTPRB | 0.528 | 41 |
| 7907830 | QSOX1 | 0.528 | 26 |
| 8099982 | APBB2 | 0.527 | 43 |
| 8057620 | COL5A2 | 0.527 | 65 |
| 8044766 | INSIG2 | 0.527 | 30 |
| 8101086 | NAAA | 0.527 | 28 |
| 7972601 | NALCN | 0.527 | 54 |
| 7951485 | SLC35F2 | 0.527 | 33 |
| 8043393 | THNSL2 | 0.527 | 27 |
| 8137709 | ZFAND2A | 0.527 | 24 |
| 8086451 | HIGD1A | 0.526 | 12 |
| 8078008 | LSM3 | 0.526 | 8 |
| 8047078 | MFSD6 | 0.526 | 28 |
| 8157700 | RABGAP1 | 0.526 | 30 |
| 7898809 | EPHB2 | 0.525 | 27 |
| 8057898 | HECW2 | 0.525 | 36 |
| 7911529 | MXRA8 | 0.525 | 11 |
| 7938687 | NUCB2 | 0.525 | 32 |
| 8021623 | SERPINB7 | 0.525 | 31 |
| 8122933 | TIAM2 | 0.525 | 34 |
| 8081838 | ARHGAP31 | 0.524 | 30 |
| 8094911 | ATP10D | 0.524 | 40 |
| 8099912 | C4orf34 | 0.524 | 22 |
| 8154233 | CD274 | 0.524 | 24 |
| 8037657 | DMPK | 0.524 | 47 |
| 8129497 | EPB41L2 | 0.524 | 32 |
| 8025828 | LDLR | 0.524 | 42 |
| 8066619 | PLTP | 0.524 | 37 |
| 7998722 | SNORD60 | 0.524 | 25 |
| 8133818 | PHTF2 | 0.523 | 28 |
| 7922598 | ANGPTL1 | 0.522 | 29 |
| 8077441 | BHLHE40 | 0.522 | 28 |
| 8168470 | COX7B | 0.522 | 8 |
| 8021695 | DOK6 | 0.522 | 28 |
| 8117580 | HIST1H2AI | 0.522 | 15 |
| 8152764 | MTSS1 | 0.522 | 29 |
| 8020630 | TTC39C | 0.522 | 33 |
| 8063043 | UBE2C | 0.522 | 37 |
| 7987165 | AVEN | 0.521 | 25 |
| 8089714 | LSAMP | 0.521 | 27 |
| 8142880 | MIR182 | 0.521 | 25 |
| 8162276 | NFIL3 | 0.521 | 27 |
| 8011343 | SPATA22 | 0.521 | 29 |
| 8112940 | SSBP2 | 0.521 | 37 |
| 7950447 | XRRA1 | 0.521 | 32 |
| 7945232 | ADAMTS15 | 0.52 | 27 |
| 7900699 | CDC20 | 0.52 | 18 |
| 7902687 | CYR61 | 0.52 | 27 |
| 8138602 | DFNA5 | 0.52 | 29 |
| 8124380 | HIST1H1A | 0.52 | 24 |
| 8122734 | PPP1R14C | 0.52 | 31 |
| 7973306 | ABHD4 | 0.519 | 30 |
| 7948741 | B3GAT3 | 0.519 | 8 |
| 7912937 | PADI2 | 0.519 | 40 |
| 7985809 | ABHD2 | 0.518 | 59 |
| 8075981 | C22orf23 | 0.518 | 27 |
| 8161024 | C9orf100 | 0.518 | 25 |
| 7962146 | FAM60A | 0.518 | 8 |
| 8152280 | LRP12 | 0.518 | 27 |
| 8047161 | OBFC2A | 0.518 | 25 |
| 8145977 | PLEKHA2 | 0.518 | 31 |
| 8151935 | RPL30 | 0.518 | 20 |
| 8119974 | SLC29A1 | 0.518 | 38 |
| 8120061 | ENPP4 | 0.517 | 35 |
| 7959761 | FAM101A | 0.517 | 32 |
| 7968417 | FRY | 0.517 | 72 |
| 8128737 | MICAL1 | 0.517 | 36 |
| 8149825 | STC1 | 0.517 | 26 |
| 7958784 | ALDH2 | 0.516 | 31 |
| 8168794 | CENPI | 0.516 | 23 |
| 8112139 | IL6ST | 0.516 | 48 |
| 8009301 | PRKCA | 0.516 | 29 |
| 7987405 | RASGRP1 | 0.516 | 43 |
| 8049297 | SCARNA5 | 0.516 | 25 |
| 8097056 | SNORA24 | 0.516 | 17 |
| 7906863 | UAP1 | 0.516 | 28 |
| 8146000 | ADAM9 | 0.515 | 26 |
| 7982663 | BUB1B | 0.515 | 30 |
| 7923378 | CSRP1 | 0.515 | 33 |
| 8092578 | ETV5 | 0.515 | 34 |
| 7902227 | GADD45A | 0.515 | 25 |
| 8103728 | HMGB2 | 0.515 | 22 |
| 7925413 | MT1P2 | 0.515 | 20 |
| 8113981 | P4HA2 | 0.515 | 46 |
| 7947801 | PACSIN3 | 0.515 | 26 |
| 8050278 | PDIA6 | 0.515 | 32 |
| 8159521 | PTGDS | 0.515 | 32 |
| 7985224 | TBC1D2B | 0.515 | 6 |
| 7944375 | TRAPPC4 | 0.515 | 28 |
| 8117054 | CAP2 | 0.514 | 32 |
| 7931439 | DPYSL4 | 0.514 | 40 |
| 7929511 | ENTPD1 | 0.514 | 32 |
| 8109407 | GALNT10 | 0.514 | 22 |
| 7917516 | GBP1 | 0.514 | 31 |
| 8055130 | HS6ST1P1 | 0.514 | 31 |
| 8100541 | IGFBP7 | 0.514 | 24 |
| 8055952 | NR4A2 | 0.514 | 28 |
| 7907572 | PAPPA2 | 0.514 | 38 |
| 8092201 | TBL1XR1 | 0.514 | 18 |
| 7982597 | THBS1 | 0.514 | 26 |
| 8110392 | TMED9 | 0.514 | 15 |
| 8097058 | CEP170L | 0.513 | 8 |
| 8169473 | PLS3 | 0.513 | 44 |
| 7899016 | SDHD | 0.513 | 9 |
| 8163185 | TXN | 0.513 | 15 |
| 8145055 | BMP1 | 0.512 | 36 |
| 7932964 | C1DP1 | 0.512 | 27 |
| 7917779 | GCLM | 0.512 | 31 |
| 7925876 | PFKP | 0.512 | 33 |
| 7983527 | SEMA6D | 0.512 | 28 |
| 7982056 | SNORD115-25 | 0.512 | 15 |
| 8093997 | SORCS2 | 0.512 | 39 |
| 8122176 | TCF21 | 0.512 | 27 |
| 8121838 | TPD52L1 | 0.512 | 27 |
| 8085774 | ZNF385D | 0.512 | 26 |
| 7999834 | ARL6IP1 | 0.511 | 21 |
| 7924619 | ENAH | 0.511 | 31 |
| 8117426 | HIST1H2BH | 0.511 | 14 |
| 8043431 | IGKV1-33 | 0.511 | 18 |
| 7946579 | LYVE1 | 0.511 | 25 |
| 8173933 | PCDH19 | 0.511 | 27 |
| 7930454 | PDCD4 | 0.511 | 39 |
| 7958152 | PGAM4 | 0.511 | 84 |
| 7918426 | SLC16A4 | 0.511 | 30 |
| 7902441 | ST6GALNAC5 | 0.511 | 30 |
| 8113573 | TSSK1B | 0.511 | 25 |
| 8130422 | CNKSR3 | 0.51 | 34 |
| 8045736 | FMNL2 | 0.51 | 39 |
| 7950885 | FZD4 | 0.51 | 41 |
| 8162142 | ISCA1 | 0.51 | 9 |
| 8045664 | LYPD6B | 0.51 | 26 |
| 8156571 | MIR27B | 0.51 | 25 |
| 7907657 | RALGPS2 | 0.51 | 24 |
| 8084717 | ST6GAL1 | 0.51 | 33 |
| 8119898 | VEGFA | 0.51 | 30 |
| 8041447 | CRIM1 | 0.509 | 48 |
| 7924351 | EPRS | 0.509 | 36 |
| 8178498 | HLA-B | 0.509 | 40 |
| 8094556 | PGM2 | 0.509 | 32 |
| 8037767 | PNMAL1 | 0.509 | 36 |
| 8140971 | SAMD9L | 0.509 | 43 |
| 8076223 | SNORD43 | 0.509 | 25 |
| 7955217 | SPATS2 | 0.509 | 35 |
| 8053484 | ST3GAL5 | 0.509 | 30 |
| 8162940 | ABCA1 | 0.508 | 65 |
| 8084206 | B3GNT5 | 0.508 | 27 |
| 7958202 | CHST11 | 0.508 | 15 |
| 8058127 | CLK1 | 0.508 | 38 |
| 8160559 | DDX58 | 0.508 | 25 |
| 8166079 | EGFL6 | 0.508 | 34 |
| 8138289 | ETV1 | 0.508 | 41 |
| 7931097 | HTRA1 | 0.508 | 36 |
| 8151334 | MSC | 0.508 | 31 |
| 8149345 | OR7E154P | 0.508 | 24 |
| 8082100 | PARP14 | 0.508 | 33 |
| 8061447 | PYGB | 0.508 | 28 |
| 8127346 | RAB23 | 0.508 | 34 |
| 8149356 | USP17L1P | 0.508 | 40 |
| 8119357 | DAAM2 | 0.507 | 37 |
| 7961546 | EPS8 | 0.507 | 26 |
| 7948656 | FTH1 | 0.507 | 17 |
| 7901010 | KIF2C | 0.507 | 28 |
| 7926900 | MAP3K8 | 0.507 | 28 |
| 7897378 | PER3 | 0.507 | 38 |
| 8160040 | PTPRD | 0.507 | 52 |
| 7927799 | REEP3 | 0.507 | 24 |
| 8145555 | SCARA3 | 0.507 | 25 |
| 7984079 | TPM1 | 0.507 | 26 |
| 8169294 | COL4A5 | 0.506 | 69 |
| 8003171 | COTL1 | 0.506 | 25 |
| 8082058 | CSTA | 0.506 | 28 |
| 7980535 | DYNLL1 | 0.506 | 9 |
| 8102342 | ELOVL6 | 0.506 | 26 |
| 8155192 | GLIPR2 | 0.506 | 30 |
| 8046861 | ITGAV | 0.506 | 36 |
| 8120602 | OGFRL1 | 0.506 | 31 |
| 8000716 | SEZ6L2 | 0.506 | 45 |
| 7913869 | STMN1 | 0.506 | 34 |
| 8169015 | TCEAL7 | 0.506 | 27 |
| 7957417 | TMTC2 | 0.506 | 28 |
| 7983360 | B2M | 0.505 | 31 |
| 8068105 | BACH1 | 0.505 | 31 |
| 8053278 | FAM176A | 0.505 | 28 |
| 8105121 | GHR | 0.505 | 29 |
| 8120654 | KCNQ5 | 0.505 | 41 |
| 7995838 | MT1X | 0.505 | 15 |
| 8061357 | PAX1 | 0.505 | 28 |
| 7967030 | RNU4-1 | 0.505 | 6 |
| 8090490 | RPN1 | 0.505 | 30 |
| 7998666 | SNORA64 | 0.505 | 25 |
| 7964830 | SNORA70G | 0.505 | 24 |
| 7976814 | SNORD114-2 | 0.505 | 23 |
| 7983744 | TMOD3 | 0.505 | 30 |
| 7930181 | AS3MT | 0.504 | 25 |
| 8096808 | CCDC109B | 0.504 | 30 |
| 7971296 | EPSTI1 | 0.504 | 33 |
| 7987145 | FMN1 | 0.504 | 23 |
| 8154951 | GLULP4 | 0.504 | 14 |
| 8015387 | KRT17 | 0.504 | 19 |
| 8103695 | MFAP3L | 0.504 | 34 |
| 7925589 | SMYD3 | 0.504 | 40 |
| 8092520 | C3orf70 | 0.503 | 32 |
| 7933750 | SLC16A9 | 0.503 | 32 |
| 7952601 | ETS1 | 0.502 | 40 |
| 8105191 | PARP8 | 0.502 | 28 |
| 8004237 | RNASEK | 0.502 | 27 |
| 7995477 | RNY4P3 | 0.502 | 4 |
| 7917674 | SET | 0.502 | 5 |
| 8083709 | SMC4 | 0.502 | 36 |
| 7976810 | SNORD113-3 | 0.502 | 22 |
| 8114536 | TMEM173 | 0.502 | 36 |
| 7969428 | UCHL3 | 0.502 | 29 |
| 8151871 | CCNE2 | 0.501 | 32 |
| 8163481 | CDC26 | 0.501 | 11 |
| 8147172 | CPNE3 | 0.501 | 43 |
| 8082075 | DTX3L | 0.501 | 32 |
| 8171865 | EEF1B4 | 0.501 | 11 |
| 7932160 | FAM107B | 0.501 | 31 |
| 7926679 | KIAA1217 | 0.501 | 44 |
| 7911799 | MEGF6 | 0.501 | 46 |
| 8097417 | PHF17 | 0.501 | 42 |
| 7946812 | RPS13 | 0.501 | 8 |
| 8075857 | TST | 0.501 | 20 |
| 8012883 | HS3ST3A1 | 0.5 | 20 |
| 8111153 | MYO10 | 0.5 | 51 |
| 8167449 | PLP2 | 0.5 | 30 |
| 8129482 | SAMD3 | 0.5 | 39 |
| 8101881 | ADH1B | 0.499 | 24 |
| 7931479 | INPP5A | 0.499 | 40 |
| 8070826 | ITGB2 | 0.499 | 44 |
| 8107706 | LMNB1 | 0.499 | 31 |
| 7984771 | LOXL1 | 0.499 | 36 |
| 8142120 | NAMPTL | 0.499 | 47 |
| 8134339 | PEG10 | 0.499 | 41 |
| 7937287 | PSMD13 | 0.499 | 31 |
| 8018966 | TIMP2 | 0.499 | 31 |
| 8026513 | TPM4 | 0.499 | 15 |
| 8059350 | AP1S3 | 0.498 | 25 |
| 7948995 | ATL3 | 0.498 | 17 |
| 8083223 | C3orf58 | 0.498 | 26 |
| 8121911 | CENPW | 0.498 | 26 |
| 8161906 | GNAQ | 0.498 | 8 |
| 7899774 | HDAC1 | 0.498 | 27 |
| 8169659 | NDUFA1 | 0.498 | 24 |
| 7936567 | RAB11FIP2 | 0.498 | 35 |
| 8136078 | TSPAN33 | 0.498 | 4 |
| 7936463 | ABLIM1 | 0.497 | 35 |
| 8121095 | ANKRD6 | 0.497 | 23 |
| 8160260 | BNC2 | 0.497 | 36 |
| 8135955 | CALU | 0.497 | 24 |
| 7939546 | CD82 | 0.497 | 35 |
| 7960340 | FOXM1 | 0.497 | 36 |
| 8088958 | GBE1 | 0.497 | 40 |
| 8143899 | IQCA1L | 0.497 | 8 |
| 8106271 | NSA2 | 0.497 | 14 |
| 7950391 | PGM2L1 | 0.497 | 41 |
| 8027398 | PLEKHF1 | 0.497 | 26 |
| 7982068 | SNORD115-31 | 0.497 | 15 |
| 7939341 | CD44 | 0.496 | 52 |
| 7926127 | CELF2 | 0.496 | 30 |
| 8005661 | CYTSB | 0.496 | 43 |
| 8113250 | ERAP1 | 0.496 | 30 |
| 7985829 | FANCI | 0.496 | 51 |
| 7903777 | GSTM5 | 0.496 | 26 |
| 7932227 | NMT2 | 0.496 | 32 |
| 8078272 | NR1D2 | 0.496 | 30 |
| 7974603 | PSMA3 | 0.496 | 26 |
| 8013660 | ALDOC | 0.495 | 30 |
| 7945882 | ART5 | 0.495 | 25 |
| 8017262 | BRIP1 | 0.495 | 27 |
| 8046906 | GULP1 | 0.495 | 34 |
| 7945680 | H19 | 0.495 | 26 |
| 8077376 | ITPR1 | 0.495 | 71 |
| 8071981 | KIAA1671 | 0.495 | 19 |
| 7965112 | PAWR | 0.495 | 32 |
| 8138689 | SKAP2 | 0.495 | 34 |
| 8126428 | TRERF1 | 0.495 | 27 |
| 8005475 | TRIM16 | 0.495 | 34 |
| 7986350 | ARRDC4 | 0.494 | 27 |
| 7908496 | CFHR2 | 0.494 | 10 |
| 8089314 | IFT57 | 0.494 | 33 |
| 7945781 | PHLDA2 | 0.494 | 28 |
| 8157691 | SKA2L | 0.494 | 12 |
| 8130578 | SNORA20 | 0.494 | 24 |
| 8060344 | TRIB3 | 0.494 | 28 |
| 8136918 | ZYX | 0.494 | 31 |
| 8086517 | CDCP1 | 0.493 | 28 |
| 8050007 | PXDN | 0.493 | 34 |
| 7982048 | SNORD115-21 | 0.493 | 19 |
| 7981157 | C14orf139 | 0.492 | 23 |
| 8047097 | GLS | 0.492 | 33 |
| 8013042 | KRT17P1 | 0.492 | 23 |
| 8108683 | PCDHB2 | 0.492 | 19 |
| 8143684 | PDIA4 | 0.492 | 30 |
| 7930537 | TCF7L2 | 0.492 | 40 |
| 7947425 | CD59 | 0.491 | 32 |
| 8016390 | COPZ2 | 0.491 | 27 |
| 8168291 | ITGB1BP2 | 0.491 | 35 |
| 8096556 | METAP1 | 0.491 | 32 |
| 8062312 | MYL9 | 0.491 | 26 |
| 8075910 | RAC2 | 0.491 | 27 |
| 8151281 | TRAM1 | 0.491 | 34 |
| 8104234 | TRIP13 | 0.491 | 44 |
| 7967412 | CDK2AP1 | 0.49 | 29 |
| 7922051 | CREG1 | 0.49 | 27 |
| 7903565 | GPSM2 | 0.49 | 38 |
| 7902883 | LRRC8D | 0.49 | 32 |
| 8044143 | C2orf40 | 0.489 | 26 |
| 7986323 | GLTSCR2 | 0.489 | 7 |
| 7975076 | HSPA2 | 0.489 | 32 |
| 7929132 | PCGF5 | 0.489 | 38 |
| 7918345 | PSMA5 | 0.489 | 33 |
| 7902527 | PTGFR | 0.489 | 26 |
| 7900792 | PTPRF | 0.489 | 50 |
| 8019988 | PTPRM | 0.489 | 39 |
| 8101260 | ANTXR2 | 0.488 | 27 |
| 8117435 | BTN3A2 | 0.488 | 28 |
| 7982712 | C15orf23 | 0.488 | 25 |
| 8056102 | CD302 | 0.488 | 25 |
| 7969243 | CKAP2 | 0.488 | 46 |
| 7941460 | DRAP1 | 0.488 | 29 |
| 8061114 | DSTN | 0.488 | 21 |
| 7930304 | GSTO1 | 0.488 | 22 |
| 7974229 | KLHDC2 | 0.488 | 29 |
| 8091422 | WWTR1 | 0.488 | 21 |
| 8051814 | ZFP36L2 | 0.488 | 33 |
| 8074251 | ATP6V1E1 | 0.487 | 19 |
| 8065569 | BCL2L1 | 0.487 | 26 |
| 8145418 | CDCA2 | 0.487 | 29 |
| 8056327 | GRB14 | 0.487 | 36 |
| 7976443 | IFI27 | 0.487 | 24 |
| 8157021 | NIPSNAP3A | 0.487 | 29 |
| 7958913 | OAS2 | 0.487 | 46 |
| 8160346 | PTPLAD2 | 0.487 | 29 |
| 8091723 | RARRES1 | 0.487 | 28 |
| 8011131 | RILP | 0.487 | 26 |
| 8097282 | SPRY1 | 0.487 | 27 |
| 8156060 | TLE4 | 0.487 | 24 |
| 8029219 | TMEM145 | 0.487 | 35 |
| 8073062 | APOBEC3B | 0.486 | 13 |
| 8006779 | ARHGAP23 | 0.486 | 10 |
| 7966135 | CORO1C | 0.486 | 27 |
| 7988467 | FBN1 | 0.486 | 77 |
| 7915229 | HEYL | 0.486 | 34 |
| 8049544 | LRRFIP1 | 0.486 | 24 |
| 8131927 | MPP6 | 0.486 | 29 |
| 7904433 | PHGDH | 0.486 | 34 |
| 8092134 | PLD1 | 0.486 | 34 |
| 8000156 | SMG1 | 0.486 | 33 |
| 8165866 | STS | 0.486 | 47 |
| 8005765 | WSB1 | 0.486 | 31 |
| 8131583 | BZW2 | 0.485 | 33 |
| 8002211 | DDX28 | 0.485 | 28 |
| 7979204 | FERMT2 | 0.485 | 45 |
| 7971526 | HTR2A | 0.485 | 27 |
| 8023593 | MC4R | 0.485 | 24 |
| 7989094 | NEDD4 | 0.485 | 41 |
| 8064790 | RASSF2 | 0.485 | 24 |
| 8066262 | SNORA71D | 0.485 | 25 |
| 8114797 | SPRY4 | 0.485 | 29 |
| 8155707 | TJP2 | 0.485 | 33 |
| 8149733 | TNFRSF10B | 0.485 | 38 |
| 8104506 | TRIO | 0.485 | 70 |
| 8105506 | ZSWIM6 | 0.485 | 31 |
| 8150439 | ANK1 | 0.484 | 66 |
| 7940153 | FAM111A | 0.484 | 31 |
| 8059838 | HJURP | 0.484 | 37 |
| 7982366 | SCG5 | 0.484 | 26 |
| 8134552 | ARPC1A | 0.483 | 36 |
| 7968789 | C13orf15 | 0.483 | 25 |
| 8103922 | CASP3 | 0.483 | 26 |
| 7929750 | ENTPD7 | 0.483 | 42 |
| 7957850 | GAS2L3 | 0.483 | 24 |
| 8147503 | LAPTM4B | 0.483 | 21 |
| 7973336 | MMP14 | 0.483 | 36 |
| 8139270 | RASA4B | 0.483 | 9 |
| 8120838 | TTK | 0.483 | 48 |
| 8052947 | CYP26B1 | 0.482 | 27 |
| 7922326 | MIR214 | 0.482 | 25 |
| 7946142 | PRKCDBP | 0.482 | 23 |
| 8121794 | SMPDL3A | 0.482 | 26 |
| 8062971 | SYS1 | 0.482 | 30 |
| 8126853 | C6orf138 | 0.481 | 22 |
| 8073752 | FAM118A | 0.481 | 29 |
| 7973918 | FAM177A1 | 0.481 | 20 |
| 7999909 | GPRC5B | 0.481 | 28 |
| 8150906 | IMPAD1 | 0.481 | 18 |
| 8056943 | KIAA1715 | 0.481 | 34 |
| 8042830 | MTHFD2 | 0.481 | 31 |
| 8011759 | PFN1 | 0.481 | 19 |
| 7977507 | RPPH1 | 0.481 | 25 |
| 7957338 | SYT1 | 0.481 | 28 |
| 8098611 | TLR3 | 0.481 | 27 |
| 7983734 | TMOD2 | 0.481 | 22 |
| 8151496 | ZNF704 | 0.481 | 33 |
| 7933488 | C10orf72 | 0.48 | 31 |
| 8026106 | CALR | 0.48 | 34 |
| 8078155 | GALNTL2 | 0.48 | 40 |
| 8001531 | MT1G | 0.48 | 19 |
| 7965918 | NT5DC3 | 0.48 | 23 |
| 7968563 | RFC3 | 0.48 | 28 |
| 8092067 | RPL22L1 | 0.48 | 24 |
| 8042335 | VDAC2 | 0.48 | 48 |
| 7956785 | XPOT | 0.48 | 10 |
| 7986789 | ATP10A | 0.479 | 42 |
| 8083941 | ECT2 | 0.479 | 31 |
| 7929932 | KAZALD1 | 0.479 | 34 |
| 8163775 | MEGF9 | 0.479 | 30 |
| 7957298 | NAV3 | 0.479 | 46 |
| 8163958 | OR1N1 | 0.479 | 24 |
| 7935116 | RBP4 | 0.479 | 27 |
| 8146914 | TERF1P3 | 0.479 | 10 |
| 8122720 | ULBP2 | 0.479 | 12 |
| 8068671 | BACE2 | 0.478 | 36 |
| 8102415 | CAMK2D | 0.478 | 26 |
| 8166493 | EIF2S3 | 0.478 | 9 |
| 8143327 | PARP12 | 0.478 | 25 |
| 7944656 | SC5DL | 0.478 | 27 |
| 7982014 | SNORD115-4 | 0.478 | 12 |
| 7981990 | SNORD116-21 | 0.478 | 12 |
| 7914557 | SYNC | 0.478 | 24 |
| 8035793 | ZNF737 | 0.478 | 4 |
| 7930857 | EMX2 | 0.477 | 32 |
| 8003611 | FAM57A | 0.477 | 27 |
| 8169717 | GRIA3 | 0.477 | 42 |
| 7924603 | LBR | 0.477 | 36 |
| 7976795 | MEG3 | 0.477 | 19 |
| 8091446 | PFN2 | 0.477 | 22 |
| 8102352 | PITX2 | 0.477 | 40 |
| 8037272 | PSG3 | 0.477 | 52 |
| 7981958 | SNORD116-9 | 0.477 | 26 |
| 7972217 | SPRY2 | 0.477 | 31 |
| 7938035 | TRIM22 | 0.477 | 32 |
| 8035318 | UNC13A | 0.477 | 28 |
| 8035779 | ZNF506 | 0.477 | 6 |
| 7916727 | ITGB3BP | 0.476 | 30 |
| 8148572 | LY6E | 0.476 | 26 |
| 7935058 | MYOF | 0.476 | 60 |
| 8144153 | NCAPG2 | 0.476 | 37 |
| 7978644 | NFKBIA | 0.476 | 25 |
| 8156848 | NR4A3 | 0.476 | 42 |
| 8015445 | NT5C3L | 0.476 | 25 |
| 8086660 | PRSS46 | 0.476 | 11 |
| 8128894 | REV3L | 0.476 | 43 |
| 7975459 | SIPA1L1 | 0.476 | 30 |
| 7965964 | SLC41A2 | 0.476 | 32 |
| 7918284 | TAF13 | 0.476 | 38 |
| 8003607 | C17orf97 | 0.475 | 24 |
| 8046201 | SSB | 0.475 | 12 |
| 8088106 | TKT | 0.475 | 27 |
| 8161558 | AQP7 | 0.474 | 24 |
| 7970111 | ARHGEF7 | 0.474 | 34 |
| 7954029 | CDKN1B | 0.474 | 26 |
| 7943442 | DYNC2H1 | 0.474 | 71 |
| 8072678 | HMOX1 | 0.474 | 28 |
| 8128669 | OSTM1 | 0.474 | 33 |
| 8089249 | RPL24 | 0.474 | 21 |
| 8021181 | SCARNA17 | 0.474 | 25 |
| 8103166 | SH3D19 | 0.474 | 27 |
| 7904469 | SRGAP2 | 0.474 | 42 |
| 7988763 | TNFAIP8L3 | 0.474 | 24 |
| 7953508 | TPI1 | 0.474 | 13 |
| 7908161 | C1orf21 | 0.473 | 30 |
| 8120679 | DDX43 | 0.473 | 40 |
| 8096875 | ENPEP | 0.473 | 28 |
| 7938295 | RPL27A | 0.473 | 24 |
| 8018761 | ST6GALNAC2 | 0.473 | 30 |
| 8092095 | TNIK | 0.473 | 39 |
| 8016739 | TOB1 | 0.473 | 26 |
| 8008139 | UBE2Z | 0.473 | 27 |
| 7979813 | ZFP36L1 | 0.473 | 32 |
| 8083240 | AGTR1 | 0.472 | 22 |
| 8166219 | SYAP1 | 0.472 | 29 |
| 8161056 | TLN1 | 0.472 | 60 |
| 8015759 | VAT1 | 0.472 | 32 |
| 8096160 | ARHGAP24 | 0.471 | 34 |
| 8163733 | CDK5RAP2 | 0.471 | 52 |
| 7924450 | DUSP10 | 0.471 | 30 |
| 7910997 | EXO1 | 0.471 | 36 |
| 8030171 | FTL | 0.471 | 15 |
| 8058147 | PPIL3 | 0.471 | 26 |
| 7977879 | PSMB5 | 0.471 | 25 |
| 8100347 | SCFD2 | 0.471 | 32 |
| 7965979 | ALDH1L2 | 0.47 | 25 |
| 8083136 | ATP1B3 | 0.47 | 10 |
| 7926223 | CAMK1D | 0.47 | 28 |
| 8067279 | CTSZ | 0.47 | 30 |
| 8095907 | FRAS1 | 0.47 | 101 |
| 7947553 | LRRC4C | 0.47 | 23 |
| 8166511 | PDK3 | 0.47 | 28 |
| 8137464 | PSPH | 0.47 | 5 |
| 8054344 | RFX8 | 0.47 | 30 |
| 8007441 | RPL27 | 0.47 | 21 |
| 8130474 | SERAC1 | 0.47 | 27 |
| 7944082 | TAGLN | 0.47 | 24 |
| 8177947 | TCF19 | 0.47 | 29 |
| 8043114 | TCF7L1 | 0.47 | 44 |
| 7957467 | C12orf29 | 0.469 | 22 |
| 8166442 | FAM3C2 | 0.469 | 47 |
| 8130374 | FBXO5 | 0.469 | 30 |
| 8107133 | PAM | 0.469 | 32 |
| 7984524 | PAQR5 | 0.469 | 34 |
| 7906564 | PEA15 | 0.469 | 28 |
| 8099340 | WDR1 | 0.469 | 46 |
| 7900922 | ATP6V0B | 0.468 | 40 |
| 7947248 | KIF18A | 0.468 | 40 |
| 8094599 | KLF3 | 0.468 | 27 |
| 8084694 | MIR1248 | 0.468 | 15 |
| 7952313 | MIRLET7A2 | 0.468 | 25 |
| 8049487 | MLPH | 0.468 | 28 |
| 8043909 | NPAS2 | 0.468 | 27 |
| 8166455 | PRDX4 | 0.468 | 33 |
| 8141035 | SGCE | 0.468 | 33 |
| 8149071 | ANGPT2 | 0.467 | 28 |
| 7918552 | C1orf183 | 0.467 | 30 |
| 8162462 | C9orf129 | 0.467 | 6 |
| 8134098 | CDK14 | 0.467 | 42 |
| 7960407 | FGF6 | 0.467 | 24 |
| 8139859 | GUSB | 0.467 | 32 |
| 8116910 | HIVEP1 | 0.467 | 29 |
| 7965064 | OSBPL8 | 0.467 | 32 |
| 8139935 | TYW1B | 0.467 | 9 |
| 7966315 | ARPC3 | 0.466 | 11 |
| 8156278 | C9orf47 | 0.466 | 38 |
| 8175531 | CDR1 | 0.466 | 28 |
| 8139656 | GRB10 | 0.466 | 22 |
| 8156164 | KIF27 | 0.466 | 6 |
| 8047228 | MOBKL3 | 0.466 | 30 |
| 7939926 | OR4C16 | 0.466 | 23 |
| 7926356 | PTER | 0.466 | 24 |
| 7926836 | RAB18 | 0.466 | 33 |
| 7952339 | SNORD14C | 0.466 | 25 |
| 8153474 | TSTA3 | 0.466 | 26 |
| 8123137 | ACAT2 | 0.465 | 29 |
| 8010260 | BIRC5 | 0.465 | 36 |
| 8123678 | C6orf145 | 0.465 | 28 |
| 8120102 | CD2AP | 0.465 | 43 |
| 8150112 | GSR | 0.465 | 31 |
| 8117395 | HIST1H2BF | 0.465 | 38 |
| 8060736 | PANK2 | 0.465 | 10 |
| 8065603 | PLAGL2 | 0.465 | 23 |
| 7945573 | POLR2L | 0.465 | 22 |
| 8078270 | RPL15 | 0.465 | 12 |
| 8130720 | SFT2D1 | 0.465 | 35 |
| 7909689 | SMYD2 | 0.465 | 40 |
| 7951032 | SNORA1 | 0.465 | 14 |
| 8146216 | VDAC3 | 0.465 | 22 |
| 8113790 | MAR3 | 0.464 | 31 |
| 8121277 | AIM1 | 0.464 | 32 |
| 8074399 | CLTCL1 | 0.464 | 36 |
| 8026729 | DDA1 | 0.464 | 24 |
| 7985555 | EFTUD1 | 0.464 | 4 |
| 8116591 | FOXC1 | 0.464 | 35 |
| 8142194 | LAMB1 | 0.464 | 42 |
| 7953218 | RAD51AP1 | 0.464 | 22 |
| 8173613 | RLIM | 0.464 | 16 |
| 7939298 | CAT | 0.463 | 34 |
| 8115147 | CD74 | 0.463 | 29 |
| 7980765 | GPR68 | 0.463 | 30 |
| 8117408 | HIST1H2AE | 0.463 | 27 |
| 7924096 | NEK2 | 0.463 | 20 |
| 8113591 | PGGT1B | 0.463 | 41 |
| 8108912 | SH3RF2 | 0.463 | 36 |
| 8162216 | SHC3 | 0.463 | 32 |
| 8152759 | TATDN1 | 0.463 | 9 |
| 8038347 | TEAD2 | 0.463 | 36 |
| 8029006 | AXL | 0.462 | 30 |
| 8139203 | C7orf11 | 0.462 | 27 |
| 7958000 | CHPT1 | 0.462 | 30 |
| 8145570 | ESCO2 | 0.462 | 38 |
| 7903742 | GSTM4 | 0.462 | 25 |
| 8101945 | H2AFZ | 0.462 | 13 |
| 7919612 | HIST2H3PS2 | 0.462 | 32 |
| 7935810 | NDUFB8 | 0.462 | 29 |
| 8121814 | NKAIN2 | 0.462 | 25 |
| 8017133 | SKA2 | 0.462 | 13 |
| 7921487 | TAGLN2 | 0.462 | 11 |
| 7967563 | UBC | 0.462 | 28 |
| 8005689 | USP32 | 0.462 | 14 |
| 8100382 | CHIC2 | 0.461 | 30 |
| 8112592 | FOXD1 | 0.461 | 27 |
| 8122317 | HEBP2 | 0.461 | 36 |
| 8022176 | LAMA1 | 0.461 | 80 |
| 8057486 | PDE1A | 0.461 | 41 |
| 7938291 | SNORA3 | 0.461 | 24 |
| 7925531 | AKT3 | 0.46 | 36 |
| 8080926 | ARL6IP5 | 0.46 | 25 |
| 8135268 | EIF4B | 0.46 | 5 |
| 7956989 | MDM2 | 0.46 | 22 |
| 8155169 | RECK | 0.46 | 36 |
| 8057394 | SESTD1 | 0.46 | 42 |
| 8150698 | SNAI2 | 0.46 | 26 |
| 7967685 | STX2 | 0.46 | 32 |
| 8123864 | TFAP2A | 0.46 | 31 |
| 8115886 | THOC3 | 0.46 | 37 |
| 7908766 | TIMM17A | 0.46 | 29 |
| 8159900 | GLIS3 | 0.459 | 60 |
| 8066231 | KIAA1755 | 0.459 | 28 |
| 7919095 | NOTCH2 | 0.459 | 38 |
| 7925492 | OPN3 | 0.459 | 31 |
| 8168589 | ZNF711 | 0.459 | 26 |
| 7975598 | ACOT1 | 0.458 | 12 |
| 8092765 | C3orf59 | 0.458 | 27 |
| 8102328 | CFI | 0.458 | 33 |
| 7923516 | CYB5R1 | 0.458 | 30 |
| 8109712 | HMMR | 0.458 | 40 |
| 8122860 | MYCT1 | 0.458 | 25 |
| 8100464 | NMU | 0.458 | 28 |
| 8043276 | PTCD3 | 0.458 | 25 |
| 8029969 | SEPW1 | 0.458 | 26 |
| 7982088 | SNORD115-41 | 0.458 | 14 |
| 8091757 | TRIM59 | 0.458 | 29 |
| 8000375 | ARHGAP17 | 0.457 | 33 |
| 8042211 | B3GNT2 | 0.457 | 32 |
| 8021685 | CCDC102B | 0.457 | 31 |
| 7983867 | CGNL1 | 0.457 | 30 |
| 8159142 | COL5A1 | 0.457 | 73 |
| 8130499 | DYNLT1 | 0.457 | 19 |
| 8007446 | IFI35 | 0.457 | 26 |
| 8137526 | INSIG1 | 0.457 | 30 |
| 8121768 | PKIB | 0.457 | 23 |
| 8055220 | POTEF | 0.457 | 8 |
| 8113726 | PPIC | 0.457 | 25 |
| 8160238 | PSIP1 | 0.457 | 20 |
| 7955502 | SCN8A | 0.457 | 38 |
| 8006531 | SLFN5 | 0.457 | 37 |
| 7951036 | SNORD5 | 0.457 | 25 |
| 8098870 | SPON2 | 0.457 | 32 |
| 8075106 | TPST2 | 0.457 | 29 |
| 8095986 | ANXA3 | 0.456 | 30 |
| 7952526 | CDON | 0.456 | 31 |
| 8065412 | CST1 | 0.456 | 8 |
| 8112980 | EDIL3 | 0.456 | 30 |
| 7992828 | IL32 | 0.456 | 25 |
| 7971565 | LPAR6 | 0.456 | 27 |
| 7901788 | NFIA | 0.456 | 35 |
| 8059345 | SCG2 | 0.456 | 27 |
| 7960865 | SLC2A3 | 0.456 | 19 |
