# Supplementary material for: Differential Expression of Extracellular Matrix-Mediated Pathways in Single-Suture Craniosynostosis
Source: PLoS One. 2011 Oct 19;6(10):e26557. doi: 10.1371/journal.pone.0026557 (PMC3197523; doi:10.1371/journal.pone.0026557)
Supplement: Table S2 — Changes in gene expression considered to be significant and large in at least one form of single-suture craniosynostosis compared to controls. (DOC) [file pone.0026557.s003.doc]

**Table S2: Changes in gene expression considered to be significant and large in at least one form of single-suture craniosynostosis compared to control**

| probeID | coronal_control  (log2 fold change) | coronal_control  (p value) | metopic_control  (log2 fold change) | metopic_control  (p value) | sagittal_control  (log2 fold change) | sagittal_control  (p value) | all_control  (log2 fold change) | all_control  (p value) | Gene Symbol |
| --- | --- | --- | --- | --- | --- | --- | --- | --- | --- |
| 7899627 | -0.87 | 0.00 | -0.98 | 0.00 | -0.51 | 0.02 | -0.62 | 0.00 | TINAGL1 |
| 7959102 | -0.86 | 0.00 | -0.78 | 0.00 | -0.46 | 0.01 | -0.52 | 0.00 | HSPB8 |
| 7999674 | -0.76 | 0.00 | -0.73 | 0.00 | -0.42 | 0.05 | -0.50 | 0.02 | MYH11 |
| 7985786 | -0.62 | 0.02 | -0.64 | 0.01 | -0.37 | 0.11 | -0.50 | 0.03 | ACAN |
| 8015366 | -0.39 | 0.09 | -0.66 | 0.00 | -0.40 | 0.05 | -0.49 | 0.01 | KRT14 |
| 8172425 | -0.19 | 0.35 | -0.30 | 0.14 | -0.59 | 0.00 | -0.47 | 0.01 | SLC38A5 |
| 7951271 | -0.81 | 0.03 | -0.51 | 0.18 | -0.52 | 0.12 | -0.47 | 0.16 | MMP1 |
| 8099850 | -0.62 | 0.00 | -0.57 | 0.00 | -0.35 | 0.00 | -0.45 | 0.00 | TMEM156 |
| 8001784 | -0.68 | 0.00 | -0.68 | 0.00 | -0.34 | 0.02 | -0.44 | 0.00 | CDH8 |
| 8100578 | -0.57 | 0.00 | -0.59 | 0.00 | -0.40 | 0.01 | -0.44 | 0.00 | EPHA5 |
| 8020779 | -0.64 | 0.00 | -0.53 | 0.00 | 0.00 | 0.98 | -0.42 | 0.01 | DSG2 |
| 7921916 | -0.98 | 0.01 | -0.98 | 0.01 | 0.10 | 0.75 | -0.41 | 0.19 | RGS5 |
| 8151816 | -0.66 | 0.00 | -0.64 | 0.00 | -0.26 | 0.16 | -0.39 | 0.03 | GEM |
| 8100808 | -0.42 | 0.13 | -0.61 | 0.02 | -0.29 | 0.23 | -0.37 | 0.12 | SULT1E1 |
| 7912692 | -0.65 | 0.00 | -0.43 | 0.01 | -0.11 | 0.45 | -0.32 | 0.03 | HSPB7 |
| 8085138 | -0.69 | 0.00 | -0.38 | 0.08 | -0.22 | 0.26 | -0.32 | 0.10 | OXTR |
| 8081235 | -0.67 | 0.00 | -0.47 | 0.00 | -0.16 | 0.27 | -0.27 | 0.06 | COL8A1 |
| 7957140 | -0.84 | 0.01 | -0.73 | 0.02 | 0.02 | 0.95 | -0.26 | 0.36 | LGR5 |
| 8126798 | -0.75 | 0.01 | -0.74 | 0.01 | -0.16 | 0.55 | -0.25 | 0.33 | GPR116 |
| 8097449 | -0.68 | 0.03 | -0.49 | 0.11 | 0.15 | 0.59 | -0.18 | 0.50 | PCDH10 |
| 8133876 | -0.74 | 0.04 | -0.32 | 0.35 | -0.20 | 0.52 | -0.17 | 0.58 | CD36 |
| 8097773 | -0.48 | 0.03 | -0.61 | 0.00 | 0.18 | 0.34 | -0.09 | 0.62 | MAB21L2 |
| 8152617 | 0.71 | 0.03 | 0.65 | 0.04 | -0.25 | 0.38 | 0.16 | 0.56 | HAS2 |
| 8174201 | 0.70 | 0.04 | 0.32 | 0.35 | 0.06 | 0.84 | 0.18 | 0.54 | BEX1 |
| 8150901 | 0.64 | 0.04 | 0.19 | 0.53 | 0.01 | 0.98 | 0.19 | 0.48 | PENK |
| 7904158 | 0.59 | 0.00 | 0.37 | 0.06 | 0.23 | 0.21 | 0.25 | 0.16 | OLFML3 |
| 7923547 | 0.62 | 0.03 | 0.63 | 0.02 | -0.12 | 0.62 | 0.26 | 0.29 | CHI3L1 |
| 8151369 | -0.23 | 0.48 | -0.38 | 0.23 | 0.84 | 0.00 | 0.28 | 0.32 | C8orf84 |
| 7909730 | 0.75 | 0.00 | 0.67 | 0.00 | 0.00 | 0.99 | 0.30 | 0.11 | KCNK2 |
| 7957452 | 1.37 | 0.00 | 1.35 | 0.00 | -0.30 | 0.17 | 0.32 | 0.13 | ALX1 |
| 8022283 | -0.05 | 0.85 | -0.01 | 0.97 | 0.61 | 0.01 | 0.33 | 0.14 | FAM38B |
| 7997642 | 0.61 | 0.02 | 0.31 | 0.24 | 0.15 | 0.52 | 0.33 | 0.15 | CRISPLD2 |
| 8102587 | 0.71 | 0.00 | 0.45 | 0.05 | 0.24 | 0.24 | 0.36 | 0.08 | C4orf31 |
| 8128991 | 0.73 | 0.00 | 0.35 | 0.11 | 0.25 | 0.20 | 0.38 | 0.05 | LAMA4 |
| 7920165 | 0.88 | 0.01 | 0.58 | 0.07 | 0.33 | 0.25 | 0.39 | 0.16 | FLG |
| 8045688 | 0.18 | 0.48 | 0.20 | 0.43 | 0.68 | 0.00 | 0.40 | 0.07 | TNFAIP6 |
| 8109752 | 0.21 | 0.35 | 0.40 | 0.07 | 0.70 | 0.00 | 0.40 | 0.04 | ODZ2 |
| 7898693 | 0.19 | 0.30 | 0.42 | 0.02 | 0.62 | 0.00 | 0.41 | 0.01 | ALPL |
| 8152522 | 0.90 | 0.00 | 0.66 | 0.01 | 0.20 | 0.34 | 0.41 | 0.05 | ENPP2 |
| 8142471 | 1.17 | 0.00 | 0.44 | 0.01 | 0.12 | 0.41 | 0.41 | 0.01 | WNT2 |
| 8099132 | 0.04 | 0.83 | 0.41 | 0.03 | 0.62 | 0.00 | 0.41 | 0.01 | CYTL1 |
| 8148070 | 0.25 | 0.45 | 0.20 | 0.53 | 0.61 | 0.03 | 0.45 | 0.11 | COL14A1 |
| 8021081 | 0.87 | 0.01 | 1.25 | 0.00 | 0.02 | 0.94 | 0.53 | 0.06 | SLC14A1 |
| 7972239 | 0.52 | 0.06 | 0.56 | 0.04 | 0.66 | 0.01 | 0.60 | 0.01 | SLITRK6 |
| 8003667 | 1.03 | 0.00 | 0.83 | 0.00 | 0.37 | 0.02 | 0.62 | 0.00 | SERPINF1 |
| 7938225 | 0.41 | 0.23 | 0.68 | 0.04 | 0.60 | 0.05 | 0.62 | 0.03 | OLFML1 |
| 8139087 | 1.08 | 0.00 | 0.76 | 0.00 | 0.66 | 0.00 | 0.66 | 0.00 | SFRP4 |
| 7903358 | 0.93 | 0.00 | 0.72 | 0.00 | 1.04 | 0.00 | 0.75 | 0.00 | VCAM1 |
| 7983630 | 1.01 | 0.01 | 0.91 | 0.02 | 0.91 | 0.01 | 0.89 | 0.01 | FGF7 |
