# Supplementary material for: Differential Expression of Extracellular Matrix-Mediated Pathways in Single-Suture Craniosynostosis
Source: PLoS One. 2011 Oct 19;6(10):e26557. doi: 10.1371/journal.pone.0026557 (PMC3197523; doi:10.1371/journal.pone.0026557)
Supplement: Table S3 — Identification of significant KEGG pathways associated with craniosynostosis-related gene expression. (DOC) [file pone.0026557.s004.doc]

**Table S3: Identification of significant KEGG pathways associated with craniosynostosis-related gene expression**

| KEGG Pathway | p value | Genes |
| --- | --- | --- |
| Focal adhesion | 9E-13 | TLN1, PDGFA, PDGFC, PDGFD, ZYX, SHC3, COL11A1, AKT3, SHC4, PRKCA, EGFR, ACTN4, ROCK2, ACTN1, FLNC, FLNB, FLNA, VEGFC, CCND1, CCND2, VEGFA, PDGFRA, PDGFRB, LAMC2, CAV2, CAV1, TNC, COL3A1, ITGA11, ITGA10, ITGB3, MYL9, IGF1R, LAMB2, RAC2, ITGAV, COMP, COL6A3, COL6A1, LAMB1, THBS1, THBS2, THBS3, ACTB, COL4A2, FLT1, COL4A1, MET, ITGA1, IGF1, ITGA2, ITGA3, HGF, ITGA4, BIRC3, COL5A2, COL5A1, LAMA2, LAMA1, LAMA4, LAMA3, ITGA6, ITGA5, LAMA5, ITGA8, ITGA7, MYLK |
| ECM-receptor interaction | 1E-12 | TNC, COL3A1, ITGA11, ITGA10, ITGB3, SDC4, SDC2, HMMR, CD47, LAMB2, CD44, ITGAV, COMP, COL6A3, COL6A1, LAMB1, THBS1, COL11A1, THBS2, THBS3, COL4A2, COL4A1, ITGA1, ITGA2, ITGA3, ITGA4, COL5A2, COL5A1, LAMA2, LAMA1, LAMA4, LAMA3, CD36, ITGA6, LAMA5, ITGA5, ITGA8, ITGA7, LAMC2 |
| Arrhythmogenic right ventricular cardiomyopathy (ARVC) | 2E-08 | ITGA11, GJA1, ITGA10, ITGB3, CDH2, CACNB4, TCF7L2, TCF7L1, DES, DMD, ITGAV, ACTB, CACNA2D1, SLC8A1, ACTN4, ITGA1, ITGA2, ACTN1, ITGA3, ITGA4, CACNA2D3, LAMA2, ITGA6, SGCG, DSG2, ITGA5, PKP2, ITGA8, ITGA7, DSP, SGCD |
| Pathways in cancer | 2E-08 | FGF6, FGF5, FGF7, PTGS2, FGF9, PDGFA, TGFB3, MMP2, GLI3, MMP1, TGFB1, TGFB2, WNT2, CCNE2, FOS, CASP3, CDKN2A, CDKN2B, SLC2A1, HHIP, RARB, FGF1, MYC, AKT3, PRKCA, EGFR, PLD1, CDK6, MECOM, DAPK1, VEGFC, CCND1, HIF1A, VEGFA, PDGFRA, PDGFRB, MDM2, LAMC2, GSTP1, FGFR2, WNT5A, WNT5B, KITLG, NFKBIA, BCL2L1, KIT, TCF7L2, TCF7L1, IGF1R, LAMB2, RAC2, ITGAV, LAMB1, BMP4, IL6, COL4A2, COL4A1, EPAS1, IL8, TGFBR1, TGFBR2, MET, SMAD3, IGF1, ITGA2, ITGA3, BIRC5, HGF, STAT1, BIRC3, FZD4, LAMA2, LAMA1, CBLB, LAMA4, CDKN1B, LAMA3, ITGA6, HDAC1, LAMA5, ETS1, PTCH2, FGF7P2 |
| Hypertrophic cardiomyopathy (HCM) | 1E-06 | ITGA11, TGFB3, ITGA10, ITGB3, CACNB4, TPM2, TPM1, TGFB1, TPM4, TGFB2, DES, DMD, ITGAV, ACTB, CACNA2D1, SLC8A1, IL6, ITGA1, ITGA2, IGF1, ITGA3, ITGA4, CACNA2D3, LAMA2, ITGA6, SGCG, ITGA5, ITGA8, ITGA7, SGCD |
| Dilated cardiomyopathy | 2E-06 | ADCY2, ITGA11, TGFB3, ITGA10, ITGB3, CACNB4, TPM2, TPM1, TGFB1, TPM4, TGFB2, DES, DMD, ITGAV, ACTB, CACNA2D1, SLC8A1, ITGA1, ITGA2, IGF1, ITGA3, ITGA4, CACNA2D3, LAMA2, ADCY9, ITGA6, SGCG, ITGA5, ITGA8, ITGA7, SGCD |
| TGF-beta signaling pathway | 6E-06 | LTBP1, GDF6, GDF5, FST, TGFB3, DCN, TGFB1, TGFB2, CDKN2B, COMP, THBS1, MYC, THBS2, THBS3, PITX2, BMP4, ROCK2, SMAD6, TGFBR1, TGFBR2, SMAD3, ACVR2A, INHBA, ID2, ID1, SMURF2, ID4, ID3, BMP6 |
| Glutathione metabolism | 1E-05 | GSTA3, GSTT2B, PGD, GSTT1, ANPEP, GSTM5, GCLM, GSTM1, GSR, GGT5, GSTM3, GSTM4, RRM2, GPX4, GPX7, GSTO1, SMS, MGST1, GSTP1, MGST2 |
| Small cell lung cancer | 8E-05 | PTGS2, NFKBIA, BCL2L1, CCNE2, LAMB2, CDKN2B, ITGAV, RARB, LAMB1, MYC, AKT3, COL4A2, COL4A1, ITGA2, CDK6, ITGA3, BIRC3, LAMA2, LAMA1, LAMA4, CCND1, CDKN1B, LAMA3, ITGA6, LAMA5, LAMC2 |
| Regulation of actin cytoskeleton | 1E-04 | FGF6, FGF5, ENAH, FGF7, PDGFA, FGF9, TIAM2, RRAS, PDGFC, MSN, PDGFD, FGF1, EGFR, ACTN4, ARHGEF7, ROCK2, ACTN1, MYH9, ARPC1A, PDGFRA, PDGFRB, FGFR2, DIAPH3, ITGA11, ITGA10, BDKRB1, ITGB2, ITGB3, BDKRB2, MYL9, PFN1, PFN2, EZR, ARPC3, RAC2, ITGAV, ACTB, ITGA1, ITGA2, IGF2, ITGA3, ITGA4, NCKAP1, ITGA6, ITGA5, ITGA8, ITGA7, MYLK, FGF7P2, MYH10, F2R |
| p53 signaling pathway | 5E-04 | CDK1, IGF1, CDK6, PMAIP1, SESN3, CCNB1, CCNE2, CCND1, CASP3, CCNB2, CDKN2A, TNFRSF10B, CCND2, CD82, RRM2, SERPINE1, MDM2, PERP, THBS1, IGFBP3, GADD45A |
| Cell adhesion molecules (CAMs) | 6E-04 | PVR, OCLN, CADM1, ITGB2, NEO1, CLDN11, CDH2, SDC4, SDC2, ALCAM, VCAM1, ITGAV, PVRL3, CNTNAP2, NEGR1, ICAM1, PTPRM, PTPRF, NRXN3, NLGN1, NFASC, ITGA4, HLA-B, PDCD1LG2, NCAM1, NCAM2, ITGA6, ITGA8, CD274, CLDN1, VCAN, JAM2, JAM3 |
| Axon guidance | 9E-04 | ABLIM1, NRP1, PLXNA2, ABLIM3, PPP3R1, LRRC4C, CXCL12, EPHB2, SEMA5A, SEMA5B, UNC5B, RAC2, SEMA7A, SEMA3D, SEMA3C, SEMA3B, SEMA3A, RASA1, NGEF, ROCK2, EFNB2, MET, NTN4, DPYSL2, SLIT2, EPHA2, SLIT3, EPHA5, EPHA4, SEMA6D, EFNA5, SRGAP2 |
| Metabolism of xenobiotics by cytochrome P450 | 2E-03 | GSTA3, GSTT2B, CYP1B1, EPHX1, ADH1B, GSTT1, GSTM5, AKR1C3, GSTM1, AKR1C2, GSTM3, GSTM4, AKR1C4, ALDH1A3, GSTO1, MGST1, GSTP1, MGST2 |
| Melanoma | 2E-03 | EGFR, FGF6, FGF5, FGF7, PDGFA, FGF9, MET, IGF1, CDK6, HGF, IGF1R, CCND1, CDKN2A, PDGFRA, MDM2, PDGFRB, PDGFC, PDGFD, FGF1, FGF7P2, AKT3 |
| Gap junction | 3E-03 | PRKCA, EGFR, CDK1, ADCY2, TUBB2B, PDGFA, TUBB2C, GJA1, LPAR1, ITPR3, PRKG1, ITPR1, PLCB4, GNAQ, ADCY9, PDGFRA, PDGFRB, GUCY1B3, PDGFC, PDGFD, PLCB1, TUBA1B, HTR2A |
| Complement and coagulation cascades | 4E-03 | PLAT, A2M, SERPING1, BDKRB1, C1R, BDKRB2, C1S, PLAUR, CD55, THBD, F3, CD59, SERPINE1, CFH, TFPI, CFI, PROS1, PLAU, F2R |
| Chronic myeloid leukemia | 5E-03 | TGFBR1, TGFBR2, TGFB3, NFKBIA, SMAD3, CDK6, BCL2L1, MECOM, TGFB1, TGFB2, CBLB, CCND1, CDKN2A, CDKN1B, HDAC1, MDM2, SHC3, MYC, AKT3, SHC4 |
| Glycolysis / Gluconeogenesis | 5E-03 | LDHA, ALDOC, HK2, PFKP, ADH1B, ALDH3A2, TPI1P1, PGM2, ALDH7A1, TPI1, ALDH1B1, PGAM4, ALDH1A3, ALDH2, ENO2, PGK1, GAPDH, ENO1 |
| MAPK signaling pathway | 7E-03 | FGF6, FGF5, FGF7, FGF9, PDGFA, TGFB3, TGFB1, TGFB2, FOS, CASP3, BDNF, MAP3K8, IL1B, RRAS, FGF1, MYC, AKT3, PRKCA, EGFR, MECOM, FLNC, FLNB, DDIT3, FLNA, PDGFRA, HSPB1, PDGFRB, STMN1, GADD45A, NGF, FGFR2, IL1R1, PPP3R1, DUSP10, CACNB4, HSPA2, RAC2, RASGRP1, MAP3K1, RASA1, HSPA8, CACNA2D1, NTF3, TGFBR1, TGFBR2, NR4A1, CACNA2D3, DUSP5, PLA2G4A, DUSP1, NTRK2, FGF7P2, DUSP6 |
| Calcium signaling pathway | 7E-03 | GNA14, CCKAR, ADCY2, PHKA1, PPP3R1, OXTR, BDKRB1, BDKRB2, ATP2B1, EDNRA, AGTR1, ATP2B4, PLCB4, PDE1C, PDE1A, CAMK2D, PLCB1, EGFR, PRKCA, SLC8A1, SLC25A4, BST1, SPHK1, PTGFR, ITPR3, VDAC2, VDAC3, ITPR1, GNAL, PLCE1, ADCY9, GNAQ, PDGFRA, PDGFRB, MYLK, HTR2A, F2R |
| Colorectal cancer | 8E-03 | EGFR, TGFBR1, MET, TGFBR2, TGFB3, SMAD3, BIRC5, FZD4, TCF7L2, TCF7L1, TGFB1, TGFB2, FOS, IGF1R, CASP3, CCND1, RAC2, PDGFRA, PDGFRB, MYC, AKT3 |
| Cell cycle | 9E-03 | TGFB3, TTK, PTTG1, TGFB1, TGFB2, CCNE2, CDKN2A, CDKN2B, BUB1, CCNA2, MYC, CDC6, CDK1, SMAD3, CDK6, CDC20, CDC26, CCNB1, CCND1, CDKN1B, CCNB2, MAD2L1, HDAC1, PLK1, CCND2, BUB1B, MDM2, GADD45A |
