# Supplementary material for: Differential Expression of Extracellular Matrix-Mediated Pathways in Single-Suture Craniosynostosis
Source: PLoS One. 2011 Oct 19;6(10):e26557. doi: 10.1371/journal.pone.0026557 (PMC3197523; doi:10.1371/journal.pone.0026557)
Supplement: Table S4 — Genes identified in the dataset related to ECM-mediated focal adhesion with significant changes in expression between cases and controls. (DOC) [file pone.0026557.s005.doc]

**Table S4: Genes identified in the dataset related to ECM-mediated focal adhesion with significant changes in expression between cases and controls**

| Component of focal adhesion KEGG pathway | Component of ECM-interaction KEGG pathway | probeID | coronal_control  (log2 fold change) | coronal_control  (p value) | metopic_control  (log2 fold change) | metopic_control  (p value) | sagittal_control  (log2 fold change) | sagittal_control  (p value) | all_control  (log2 fold change) | all_control  (p value) | Gene.Symbol |
| --- | --- | --- | --- | --- | --- | --- | --- | --- | --- | --- | --- |
| x |  | 8028524 | -0.15 | 0.00 | -0.15 | 0.00 | -0.09 | 0.04 | -0.11 | 0.01 | ACTN4 |
| x |  | 8135587 | -0.18 | 0.02 | -0.08 | 0.28 | -0.04 | 0.58 | -0.06 | 0.33 | CAV2 |
| x |  | 7950012 | 0.02 | 0.61 | 0.08 | 0.03 | 0.03 | 0.42 | 0.07 | 0.05 | CCND1 |
|  | x | 8133876 | -0.74 | 0.04 | -0.32 | 0.35 | -0.20 | 0.52 | -0.17 | 0.58 | CD36 |
|  | x | 7939341 | -0.04 | 0.54 | 0.05 | 0.47 | -0.14 | 0.02 | -0.06 | 0.29 | CD44 |
| x | x | 7918064 | -0.29 | 0.13 | -0.04 | 0.84 | 0.41 | 0.01 | 0.13 | 0.40 | COL11A1 |
| x | x | 7972750 | -0.43 | 0.04 | -0.50 | 0.02 | 0.12 | 0.50 | -0.16 | 0.39 | COL4A1 |
| x | x | 7970033 | -0.38 | 0.02 | -0.43 | 0.01 | 0.04 | 0.78 | -0.16 | 0.27 | COL4A2 |
| x | x | 8057620 | -0.10 | 0.08 | -0.13 | 0.01 | 0.05 | 0.26 | -0.05 | 0.33 | COL5A2 |
| x | x | 8059905 | 0.17 | 0.03 | 0.10 | 0.18 | -0.03 | 0.64 | 0.05 | 0.44 | COL6A3 |
| x |  | 8135990 | -0.23 | 0.00 | -0.16 | 0.04 | -0.13 | 0.06 | -0.16 | 0.02 | FLNC |
| x |  | 7970763 | -0.53 | 0.00 | -0.41 | 0.00 | -0.34 | 0.01 | -0.40 | 0.00 | FLT1 |
| x |  | 7965873 | 0.55 | 0.04 | 0.35 | 0.17 | 0.40 | 0.08 | 0.40 | 0.08 | IGF1 |
| x | x | 7904761 | -0.14 | 0.44 | -0.12 | 0.50 | -0.32 | 0.05 | -0.32 | 0.05 | ITGA10 |
| x | x | 7989985 | 0.03 | 0.75 | 0.04 | 0.71 | 0.25 | 0.01 | 0.13 | 0.13 | ITGA11 |
| x | x | 8008237 | -0.51 | 0.00 | -0.21 | 0.13 | 0.01 | 0.91 | -0.19 | 0.13 | ITGA3 |
| x | x | 8046695 | -0.36 | 0.04 | -0.13 | 0.46 | 0.21 | 0.18 | 0.06 | 0.69 | ITGA4 |
| x | x | 7963880 | -0.39 | 0.04 | -0.02 | 0.89 | 0.05 | 0.78 | -0.03 | 0.84 | ITGA7 |
| x | x | 8022176 | 0.22 | 0.02 | 0.24 | 0.01 | 0.06 | 0.48 | 0.10 | 0.20 | LAMA1 |
| x | x | 8121949 | -0.53 | 0.00 | -0.38 | 0.03 | -0.24 | 0.11 | -0.34 | 0.02 | LAMA2 |
| x | x | 8128991 | 0.73 | 0.00 | 0.35 | 0.11 | 0.25 | 0.20 | 0.38 | 0.05 | LAMA4 |
| x | x | 8067409 | -0.34 | 0.01 | -0.22 | 0.06 | 0.06 | 0.56 | -0.10 | 0.31 | LAMA5 |
| x | x | 7908072 | -0.21 | 0.34 | -0.14 | 0.52 | -0.37 | 0.05 | -0.31 | 0.09 | LAMC2 |
| x |  | 8137670 | -0.41 | 0.00 | -0.33 | 0.01 | 0.04 | 0.75 | -0.10 | 0.37 | PDGFA |
| x |  | 8103399 | 0.44 | 0.00 | 0.43 | 0.00 | 0.09 | 0.43 | 0.23 | 0.04 | PDGFC |
|  | x | 8066513 | -0.01 | 0.92 | 0.02 | 0.85 | -0.22 | 0.01 | -0.13 | 0.12 | SDC4 |
| x |  | 7988563 | -0.56 | 0.00 | -0.37 | 0.01 | -0.23 | 0.08 | -0.27 | 0.04 | SHC4 |
| x | x | 8130867 | 0.09 | 0.18 | -0.04 | 0.57 | 0.20 | 0.00 | 0.12 | 0.04 | THBS2 |
